# Integrated gene set analysis for microRNA studies

**DOI:** 10.1093/bioinformatics/btw334

**Published:** 2016-06-20

**Authors:** Francisco Garcia-Garcia, Joaquin Panadero, Joaquin Dopazo, David Montaner

**Affiliations:** ^1^Computational Genomics Department, Centro de Investigacion Principe Felipe (CIPF), Valencia, Spain; ^2^Genometra S.L., Valencia, Spain; ^3^Bioinformatics of Rare Diseases (BIER), CIBER de Enfermedades Raras (CIBERER), Valencia, Spain; ^4^Functional Genomics Node, (INB) at CIPF, Valencia, Spain

## Abstract

**Motivation**: Functional interpretation of miRNA expression data is currently done in a three step procedure: select differentially expressed miRNAs, find their target genes, and carry out gene set *overrepresentation analysis*. Nevertheless, major limitations of this approach have already been described at the gene level, while some newer arise in the miRNA scenario.

Here, we propose an enhanced methodology that builds on the well-established *gene set analysis* paradigm. Evidence for differential expression at the miRNA level is transferred to a gene *differential inhibition* score which is easily interpretable in terms of gene sets or pathways. Such *transferred indexes* account for the additive effect of several miRNAs targeting the same gene, and also incorporate cancellation effects between cases and controls. Together, these two desirable characteristics allow for more accurate modeling of regulatory processes.

**Results**: We analyze high-throughput sequencing data from 20 different cancer types and provide exhaustive reports of gene and Gene Ontology-term deregulation by miRNA action.

**Availability and Implementation**: The proposed methodology was implemented in the Bioconductor library mdgsa. http://bioconductor.org/packages/mdgsa. For the purpose of reproducibility all of the scripts are available at https://github.com/dmontaner-papers/gsa4mirna

**Contact**: david.montaner@gmail.com

**Supplementary information:** Supplementary data are available at *Bioinformatics* online.

## 1 Introduction

MicroRNAs (miRNAs) are small non-coding RNA molecules which participate in post-transcriptional gene regulation (He and Hannon, 2004). They bind to target mRNAs with partial complementarity, causing translational repression or target degradation (Wei *et al.*, 2013). Aberrant miRNAs expression has been reported to be linked to disease (Jiang *et al.*, 2009) and so many genomic experiments are now being conducted with the aim of clarifying the relationship between miRNA levels and phenotype. These experiments generally use microarrays or high-throughput sequencing to record miRNA expression between different biological conditions, followed by differential-expression analysis to evaluate the association of each miRNA to phenotype. It is common in such analyses to first select the significantly different miRNAs, and then explore their target genes to infer possible functional consequences of the deregulation of these miRNAs. Gene function databases, such as the Gene Ontology (GO) (Ashburner *et al.*, 2000), KEGG (Kanehisa and Goto, 2000) or Reactome (Joshi-Tope *et al.*, 2005) are commonly used in this second step. Some authors prefer to first annotate miRNAs onto the functions of their target genes, and then do the functional interpretation at the miRNA level (Bleazard *et al.*, 2015; Godard and van Eyll, 2015). Despite being less instinctive or intuitive, this approach has been shown to reduce the effect of biased database information. This two-step paradigm, known as *over representation analysis* (ORA), has been extensively used in gene expression experiments and is now, the exclusive method used for miRNA functional profiling.

But even in the gene expression context, ORA approaches have been legitimately criticized and some major drawbacks have been described (Dopazo, 2009; Khatri *et al.*, 2012). Most concerning of these disadvantages is the loss of information caused by using only a few genes and the egalitarian treatment of these selected genes, a problem that arises again in the miRNA scenario. In differential gene-expression analyses for instance, ORA only considers genes which show large expression differences, whereas small changes in functionally related *gene sets* may be more relevant to the underlying biology. Similar biases occur when analyzing miRNA expression data, but in this case the effect is doubled. On one hand, some genes may be regulated by a big change in a single miRNA. If this occurs in an experiment, the miRNA will be identified as differentially expressed and therefore ORA can be used, with the above mentioned limitations. On the other hand, some other less-robust gene deregulations may go unnoticed because the miRNAs causing them do not appear among the most differentially expressed candidates, thus, in such cases the combined *gene set* effect will be missed. Furthermore, genes can also be inhibited by the additive effect of several small miRNA changes (Doxakis, 2010; Papapetrou *et al.*, 2010). This scenario is common but is usually neglected in the ORA because the causative miRNAs are unlikely to be selected in the two-stages approach. Finally, a gene may be regulated by several miRNAs with opposite expression patterns (Bleazard *et al.*, 2015). This may induce compensatory effects that, presumably, are not considered by ORA approaches. As a simple example of this later situation we can think about a gene modulated by two miRNAs, one of them up regulated in experimental cases and the other up regulated in controls. The gene will be down regulated or inhibited in both conditions and hence, is irrelevant for case-control comparison. Despite this, ORA algorithms are likely to identify such genes as relevant in the comparison, because their regulatory miRNAs will have been selected in the differential-expression step of the analysis (Godard and van Eyll, 2015).

Thus, the application ORA methodology intrinsically implies a relatively naive understanding of biology. In the context of gene expression, the limitations of ORA have already been surpassed by *gene set analysis* (GSA) methods (Mootha *et al.*, 2003). GSA approaches which can successfully model the importance of weaker, but coordinated changes in sets of functionally related genes, therefore reinforcing genomic data interpretation. But, even though GSA methods have been available for a long time for gene-based experiments, to our knowledge, no GSA-like methodologies have so far been proposed for functional profiling of miRNA measurements. This lack of GSA-style applications to miRNA data is not really surprising for two reasons: first, functional annotation is normally attached to genes, thus, in order to interpret miRNA data (for instance in terms of GO or KEGG), scientists must first define how miRNA and database information should be linked. For this purpose, meaningful miRNA-to-gene transfer of the experimental evidence is implicitly necessary. Second, most GSA algorithms are such that the gene-level analysis and the *enrichment* steps are strongly interdependent and cannot easily be split up. Such lack of flexibility of most GSA algorithms hinders their re-implementation and usage in the miRNA context.

For instance in the classical GSEA algorithm (Subramanian *et al.*, 2005), the statistical significance of the enrichment is evaluated using a phenotype-based permutation applied to the gene-expression data matrix. Thus, the differential-expression step is carried out within the re-sampling schema, and cannot be changed without rewriting the algorithm.

In this paper, we propose a novel GSA-type methodology for functionally interpreting miRNA expression data. Taking advantage of the additive inhibitor effect that miRNAs may have on genes, we first propose a meaningful procedure for transferring miRNA differential expression evidence to the gene level via a *differential inhibition* score. Then we use logistic regression models (Montaner and Dopazo, 2010; Montaner *et al.*, 2009; Sartor *et al.*, 2009) to interpret this gene inhibition information in terms of *gene sets*.

To exemplify the applicability of our method here we analyze 20 different real datasets taken from *The Cancer Genome Atlas* project (McLendon *et al.*, 2008). Tumor samples are compared to normal tissue in a differential miRNA expression analysis and then, functional profiling in terms of GO is carried out for each of them. Several GO terms already known to be cancer related appear as deregulated in the different cancers, validating the suitability of our approach. We hope our algorithm, implemented in the R/Bioconductor package mdgsa (Montaner and Dopazo, 2010), will be useful to data analysts, but also that the extensive supplementary materials presented in this paper would constitute a valuable asset.

## 2 Materials and methods

At the time of writing this paper 32 datasets were registered in the *The Cancer Genome Atlas* project. We downloaded and analyzed 20 of these: those with miRNA expression information, measured using *Illumina HiSeq* technology (Bentley *et al.*, 2008), which contain both tumoral and healthy samples. Table 1 shows the reference for the downloaded datasets and the number of samples included in each analysis.

**Table 1.** Analyzed datasets

ID	Total	Cases	Controls	Paired	Description
BLCA	271	252	19	19	Bladder Urothelial Carcinoma
BRCA	807	720	87	86	Breast invasive carcinoma
CESC	218	215	3	3	Cervical squamous cell carcinoma
COAD	243	235	8	0	Colon adenocarcinoma
ESCA	113	102	11	11	Esophageal carcinoma
HNSC	519	475	44	43	Head and Neck squamous cell carcinoma
KICH	91	66	25	25	Kidney Chromophobe
KIRC	311	240	71	68	Kidney renal clear cell carcinoma
KIRP	245	211	34	34	Kidney renal papillary cell carcinoma
LIHC	283	233	50	49	Liver hepatocellular carcinoma
LUAD	474	428	46	39	Lung adenocarcinoma
LUSC	376	331	45	45	Lung squamous cell carcinoma
PAAD	100	96	4	4	Pancreatic adenocarcinoma
PCPG	182	179	3	3	Pheochromocytoma and Paraganglioma
PRAD	117	100	17	17	Prostate adenocarcinoma
READ	93	90	3	0	Rectum adenocarcinoma
SKCM	75	74	1	0	Skin Cutaneous Melanoma
STAD	345	306	39	39	Stomach adenocarcinoma
THCA	558	499	59	59	Thyroid carcinoma
UCEC	418	386	32	19	Uterine Corpus Endometrial Carcinoma

Columns of the table display: TCGA disease ID, the total number of samples in the analysis, the number of tumoral samples, the number of control samples (solid normal tissue), the number of paired samples available in the dataset and the cancer type.

Preprocessed miRNA expression-count matrices were downloaded from *The Cancer Genome Atlas* data portal https://tcga-data.nci.nih.gov/tcga. Differential expression analysis, comparing *primary tumor* samples to *solid normal* tissue, was carried out using an *unpaired* approach for all 20 datasets. In addition, we also performed a *paired* analysis for 17 of them: the datasets containing tumoral and normal samples from the same individual. These miRNA-level analyses were done using the Bioconductor (Gentleman *et al.*, 2004) library edgeR (Robinson *et al.*, 2010).

Thus, for each comparison, *P*-values and test statistics were obtained at the miRNA level. The p-value represents the strength of the differential miRNA expression between cases and controls, while the sign of the statistic indicates the sense, or ‘direction’, of that difference; in our case, positive statistic values indicate overexpression in cases compared to controls, and negative statistic values indicate underexpression. For each miRNA, these two quantities can be combined in a unique index, accounting for the strength and sense of the differential expression using the following transformation:

(1) r=−sign(statistic)·log(P-value)

The computed values r are comparable across different miRNAs as they represent the original *P*-values. In addition, r also retains the sign of the test statistic, preserving the information about the ‘direction’ of the overexpression. It is therefore an index that ranks the miRNAs according to their expression-level differences; from those which are more overexpressed in cases, (the ones with the highest positive values), to those which are more underexpressed in cases, (indexes which are more negative). According to the definition, miRNAs with an r index value close to zero are those with similar expression levels in both cases and controls, that is, the ones that are not differentially expressed. In this case we derived our r values using edgeR although any other statistical test, even fold changes could be used to obtain a ranking index provided that it has the above mentioned characteristics.

### 2.1 Adding the effect on genes

MicroRNA molecules regulate gene expression via complementary base-pairing (Bartel, 2004), therefore, the inhibition of certain gene must be proportional to the amount of miRNA molecules targeting it. Moreover, many different miRNAs may intercept the same gene, thus having an additive effect on its expression levels (Gusev, 2009; Lim *et al.*, 2005). Hence, the interference of a gene must be directly related to the sum of the expression levels of its binding miRNAs. When comparing biological samples, differences in miRNA expression between experimental conditions can be reflected in different gene-inhibition patterns, and the *differential inhibition* of each gene might be proportional to the sum of the expression differences of its binding miRNAs. We can express this using the formula:

(2) $$t_{i}=\sum_{j\in G_{i}}r_{j}$$

where $t_{i}$ represents the increment in the inhibition of gene i, $r_{j}$ accounts for the differential expression of miRNA j, and $G_{i}$ is the set of microRNAs targeting gene i. The utility of similar scores in summarizing the effect of several miRNAs on a given gene has been described before (Lee *et al.*, 2012; Morin *et al.*, 2008).

**Fig. 1.**
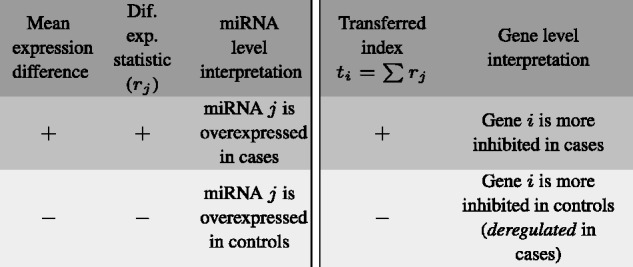
Interpretation of the differential expression statistic at miRNA level and the *transferred index* at gene level

Using Equation 2 we can ‘transfer’ the relevant information in our experiment from the miRNA to gene level, i.e. from miRNA differential-expression values to gene *differential-inhibition* estimates. Carrying out the computation for all the genes in an experimental dataset, we can derive a new *transferred index* which ranks genes according to their *differential inhibition*, caused by miRNA activity between biological conditions. Genes showing the highest *differential inhibition index* would be those more likely to be intercepted in cases, while those showing the lowest indexes should correspond to genes that are more inhibited in controls compared to cases. Genes with a *differential inhibition index* close to zero are those showing no significant differences in terms of their regulation by miRNAs. Figure 1 shows a summary of the interpretation of miRNA and gene-level results.

Here, we should note that a strong differential inhibition pattern for a gene may be due to a very big differential expression in just one of the miRNAs targeting it. But it is also likely that some of these big effects are caused by the additive effect of a particular gene being targeted by many different miRNAs, each with weaker individual differential-expression patterns between conditions.

It is also worth highlighting that, genes presenting no *differential inhibition* may be those for which none of their regulatory microRNAs are differentially expressed, but also those for which the differential expression patterns of their binding miRNAs cancel each other out by adding up to zero. For instance, in a case control experiment, the first scenario would be that none of the miRNAs targeting a given gene are differentially expressed. In which case, all the $r_{j}$ values in Equation 2 would be equal to zero, as would their sum as well as $t_{i}$ parameter. The second scenario would occur when a subset of the microRNAs targeting the gene are overexpressed, increasing the gene inhibition in cases, but another subset of miRNAs are underexpressed, increasing the inhibition in controls. Thus, both inhibition effects will then cancel each other out, resulting in no regulatory differences between cases and controls for that gene. In this second case some $r_{j}$ values will be positive and some will be negative, but their sum will yield a $t_{i}$ value close to zero.

Obviously, to implement Equation 2 or, more generally, to be able to ‘transfer’ information from the miRNAs to their target genes, the relationship between miRNAs and their gene targets must be previously defined. In this study we took this information from the *TargetScan Predicted and Conserved Targets* database (Friedman *et al.*, 2009) but any other source of similar information could be used with our software. Currently, most of the information available regarding miRNA targets is predicted by computational approaches which have limited accuracy (Selbach *et al.*, 2008) and which incorporate functional biases (Bleazard *et al.*, 2015). Thus, care should still be taken when interpreting or validating results. In any case, our method and software will remain valid and can continue to be used as this database become more curated, or should other, more-sophisticated sources become available in the near future.

It is worth noting here that Equation 2 can be easily modified to incorporate weights accounting for the quality of the miRNA-target information. Moreover, besides the knowledge aspect, weighting can also be used to improve the modeling by including extra biological information, such as the number of target sites genes have or gene expression levels, when available.

Equation 2 involves genes as miRNA targets but, as it is, it does not account for whether the genes are expressed or not. But, given that mRNA bridges miRNA functionality, if gene expression data were available alongside with miRNA levels, it would be sensible to incorporate them to the analysis. In such case, researchers may prefer to restrict the functional interpretation of the transferred miRNA index to just those genes which are effectively expressed. Equation 2 can be easily modified for such purpose by setting $t_{i}=0$ if gene i is not expressed. This alteration in the process can be done trivially using our mdgsa library (see Supplementary Materials).

### 2.2 Gene set analysis of the transferred index

In the previous section, we described how differential expression information measured at the miRNA level can be meaningfully ‘transferred’ to the gene level by computing our gene *inhibition index*.

This transferred index implies ranking the genes in such a way that gene regulation via miRNA action is easily interpretable. This gene ranking is, of course, informative on its own but it also has the advantage of being straight forward to interpret in terms of *gene sets* such as those described by the GO (Ashburner *et al.*, 2000), KEGG (Kanehisa and Goto, 2000) or Reactome (Joshi-Tope *et al.*, 2005) databases, if the appropriate *gene set analysis* method is applied.

Logistic regression models have been previously successfully used for *gene set analysis* based on a ranking statistic. (Sartor *et al.*, 2009) described how this model can be used to functionally interpret differential gene-expression studies, and (Montaner *et al.*, 2009) introduced its use in a gene-importance weighting schema. Later, (Montaner and Dopazo, 2010) developed them in the context of multiple genomic dimensions, and analyzed genomic characteristics other than the classic gene expression. More recently, (Mi *et al.*, 2012) adapted them to cope with gene-length biases in RNA-Seq studies.

Given the *ranking statistics* for the genes, t, for each functional class being studied, F, the logistic regression approach models the dependence between gene membership to the class F and the t value assigned to the gene as follows:

(3) $$\log\frac{P(g_{i}\in F)}{P(g_{i}\notin F)}=\kappa +\alpha \;t_{i}$$

When the estimated slope parameter α is significantly positive we declare the high values of the ranking t as enriched in the given function. If the α estimate is negative we say that the enrichment occurs in the lower values of the ranking t.

**Fig. 2.**
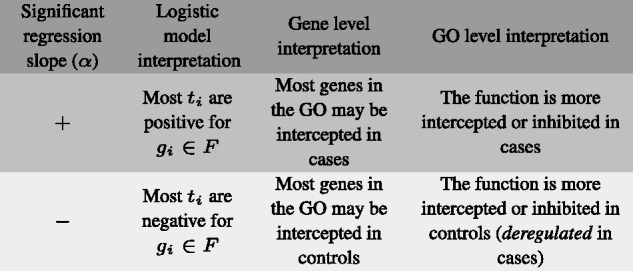
Interpretation of the logistic regression model slope parameter in terms of genes and *gene sets*

When interpreting our *transferred index*, a positive t ranking value is indicative of a certain degree of gene inhibition in the cases with respect to the controls. Hence, a positive α estimate in equation 3 indicates that genes inhibited in cases are enriched in function F. Conversely, a negative α value corresponds to an enrichment of the function in the genes which are more inhibited in controls than in cases. An α estimate which is not significantly different from zero indicates that there is no pattern of *gene set* enrichment related to the ranking. Figure 2 shows a summary of this interpretation.

Equation 2 will result in $t_{i}=0$ for genes not targeted by any miRNA, and these zeros have no major effect in Equation 3. Thus, effectively, our *gene set analysis* is bound to genes which are targeted by at least one miRNA. In ORA approaches, the use of only targeted genes has been reported as beneficial compared to other approaches which use all annotated genes as a background for testing (Bleazard *et al.*, 2015; Godard and van Eyll, 2015).

In our study we used GO (Ashburner *et al.*, 2000) terms to define our *gene sets*. Gene annotation was downloaded from the Ensembl web page http://www.ensembl.org. We analyzed the *Biological Process*, *Cellular Component* and *Molecular Function* ontologies to obtain an α estimate and its corresponding *P*-value for each GO term examined. We corrected the *P*-values for multiple testing in order to control the false discovery rate using the method from (Benjamini and Yekutieli, 2001).

**Fig. 3.**
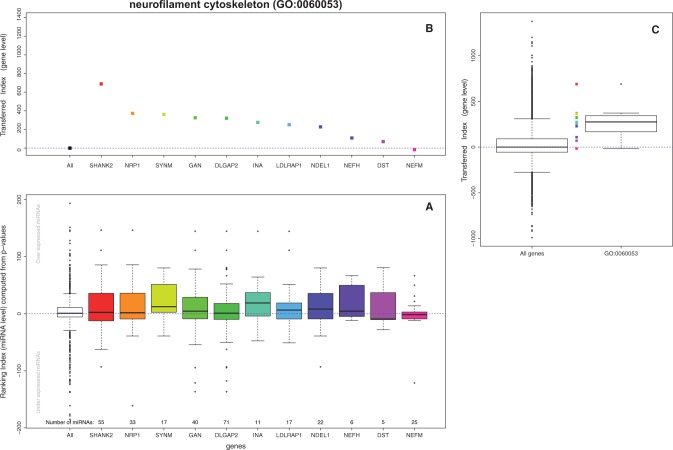
Example diagram of the analysis steps for the *neurofilament cytoskeleton* GO term (GO:0060053). Plot (**A**) represents the distribution of the ranking index computed as described in Equation 1. The white box shows the distribution for all miRNAs in the study. In our case, positive values belong to those miRNAs more expressed in tumors while the negative relate to miRNAs more expressed in controls. Each of the colored boxes represents the same index, but just for the subset of miRNAs targeting one gene in the GO. Plot (**B**) represents the gene *transferred index* introduced in Equation 2. For each of the genes in the GO term all miRNA level indexes are added up into a unique value. Each of the dots in plot B represents the gene level transferred index computed from the microRNAs represented in the boxplot underneath (plot A). Plot (**C**) displays the distribution of the *transferred index* for the whole genome (left box) and for the genes within the *neurofilament cytoskeleton* GO term (right box and dots). Here, we can appreciate how the overall distribution of the genes in the GO term is higher that the basal distribution of all genes. The logistic regression model spots this pattern and reports the GO term as enriched in tumor samples, meaning that the *neurofilament cytoskeleton* cellular component is more intercepted by miRNA action in cases than in controls

A diagram of the analysis pipeline is shown in Figure 3. Here, we present the results for the *neurofilament cytoskeleton* GO term in the paired breast invasive carcinoma (BRCA) dataset study as a worked example for our proposed algorithm.

## 3 Results and discussion

### 3.1 MicroRNA level

Differential expression analysis was carried out for each cancer type using edgeR followed by *P*-value correction to control the false discovery rate (Benjamini and Hochberg, 1995). Table 2 shows the number of up and down-regulated miRNAs in each of the cancer types for the paired and unpaired analyses. It is worth noting the large number of differentially expressed miRNAs this analysis produces, even after multiple testing correction of the *P*-values. This is due to the big differences that exist between tumor and normal samples, but also highlights the large number of miRNAs that regulate the genes expressed in one single tissue. As a consequence, interpreting the statistical results to draw biologically meaningful conclusions may be a daunting endeavor.

**Table 2.** Number of up, down and not differentially regulated miRNAS in each cancer type

	Unpaired			Paired		
ID	Down	noDif	Up	Down	noDif	Up
BLCA	128	337	353	127	343	219
BRCA	200	244	396	202	215	269
CESC	92	621	73	29	537	65
COAD	174	291	262			
ESCA	98	443	152	62	464	133
HNSC	204	285	360	164	305	222
KICH	166	297	199	217	252	169
KIRC	169	191	323	213	180	215
KIRP	221	262	295	223	242	237
LIHC	120	278	407	200	283	213
LUAD	152	292	405	130	264	259
LUSC	169	215	462	180	313	244
PAAD	23	607	11	8	606	14
PCPG	70	608	43	40	507	55
PRAD	76	429	104	38	513	31
READ	136	307	204			
SKCM	46	680	6			
STAD	152	308	356	138	307	206
THCA	218	351	257	226	347	145
UCEC	243	284	347	211	272	229

The difficulties in interpreting which of the biological functions are deregulated by miRNAs in cancer become more evident if we explore genes which are the targets of these differentially expressed miRNAs. Table 3 shows the number of genes targeted by the up and down-regulated miRNAs in each cancer type. Some saturation effects may be caused by the large number of differentially expressed miRNAs and also, by the even larger number of known target genes for each miRNA. On average, 8000 genes are targeted by up or down-regulated miRNAs, and moreover, the number of genes which are common targets of the miRNAs is very high, at around 6000 (see Table 3). In some extreme cases, more theoretical than practical, most genes in the genome could be simultaneously targeted by up and down regulated miRNAs, but unlike previous ORA approaches, our methodology is still meaningful in such cases.

**Table 3.** Number of genes targeted by the up and down regulated miRNAS

	Unpaired			Paired		
ID	Down	Common	Up	Down	Common	Up
BLCA	8345	6763	8599	8087	5955	7528
BRCA	8968	7700	9465	9305	7724	9001
CESC	7834	5201	6525	4877	3178	5431
COAD	6981	6418	9998			
ESCA	7992	5646	6959	8233	5207	6212
HNSC	9090	7496	8976	9065	7006	8013
KICH	8998	7044	8252	9594	7125	7902
KIRC	8838	7351	9056	9575	7543	8681
KIRP	9169	7388	8629	9311	7025	8267
LIHC	7466	6848	9560	8896	6851	7720
LUAD	8255	7354	9898	8150	6843	8848
LUSC	8535	7265	9447	8844	6710	8166
PAAD	3759	616	1169	1529	442	1748
PCPG	6303	4033	5295	4102	3110	5652
PRAD	7422	5932	8039	4997	1600	2374
READ	6938	6225	9672			
SKCM	5983	631	857			
STAD	8921	6761	8041	8947	6731	7855
THCA	8763	7244	8702	9064	7065	8056
UCEC	9182	7171	8436	9338	7069	8201

The Common column shows the number of genes which are targets of both, the up and down regulated miRNAs. The total number of genes which are targets of at least one miRNA is 12084.

Table 4 shows the number of GO terms associated with genes which where up and down regulated by miRNAs. As we can see, for most cancer types, all the GO terms included in the study were represented by these genes. Obviously, in this scenario, *over representation analysis* methodologies are meaningless for functional interpretation of the results. This situation is generally handled by ‘ad hoc’ methods such as increasing the cut-off *P*-value so that fewer miRNAs are called as being differentially expressed and consequently smaller groups of genes need to be interpreted. But the opposite pattern is also likely to arise in genomic studies; in the cancer case, a large number of miRNAs are expected to be differentially expressed, but we can easily imagine an experiment resulting in very few or even no differentially expressed miRNAs due, for instance, to sample size restrictions. In such cases ORA methodologies are not applicable but *gene set analysis* style methods, like the one presented here, might allow researchers to extract some meaningful conclusions from the data.

**Table 4.** Number of GO terms associated with the genes targeted by the up and down regulated miRNAs

	Unpaired			Paired		
ID	Down	Common	Up	Down	Common	Up
BLCA	5169	5169	5169	5169	5168	5168
BRCA	5169	5169	5169	5169	5169	5169
CESC	5169	5168	5168	5144	5138	5160
COAD	5168	5168	5169			
ESCA	5169	5168	5168	5169	5167	5167
HNSC	5169	5169	5169	5169	5169	5169
KICH	5169	5169	5169	5169	5169	5169
KIRC	5169	5169	5169	5169	5169	5169
KIRP	5169	5169	5169	5169	5169	5169
LIHC	5169	5169	5169	5169	5169	5169
LUAD	5169	5169	5169	5169	5169	5169
LUSC	5169	5169	5169	5169	5169	5169
PAAD	5129	4578	4590	4870	4681	4915
PCPG	5166	5161	5164	5150	5146	5165
PRAD	5169	5169	5169	5159	4981	4990
READ	5168	5168	5169			
SKCM	5169	4385	4385			
STAD	5169	5169	5169	5169	5169	5169
THCA	5169	5169	5169	5169	5169	5169
UCEC	5169	5169	5169	5169	5169	5169

Most GO terms are targeted in cases and controls at the same time as it can be seen in the *Common* column. The total number of GO terms annotated for the targeted genes is **5169**.

### 3.2 Gene level

After miRNA differential expression analysis, Equation 1 was used to summarize the *P*-values and sign statistics into a single ranking statistic. Then, Equation 2 was applied to translate this miRNA differential expression evidence into a gene *differential inhibition* scale. For each gene, this *transferred index* condenses the information about the miRNAs which target it, preserving two characteristics suitable for the functional interpretation of the experiment: it accounts for the multiple miRNA cancellation effect and it incorporates the additive effect of several small inhibitory events.

For example the *GPR162* gene is targeted by two miRNAs: *hsa-miR-22-3p* and *hsa-miR-214-3p*. In paired analysis of *kidney chromophobe* (KIRCH) carcinoma, overexpression of *hsa-miR-22-3p* was reported in tumor samples (with a *P*-value of $5.6\times 10^{-30}$) while *hsa-miR-214-3p* was underexpressed (with a confidence level of $1.8\times 10^{-29}$). Over expression indexes derived using Equation 1 where 67.34 for *hsa-miR-22-3p* and -66.61 for *hsa-miR-214-3p*, indicating that there is evidence for very similar differential expression of these two miRNAs, but in opposite ‘directions’. Hence, the gene *GPR162* must be inhibited in cases by miRNA *hsa-miR-22-3p* with the same strength that it is inhibited in controls by miRNA *hsa-miR-214-3p*. Therefore, our interpretation is that, both inhibition effects cancel each other out and so, gene *GPR162* is considered to be irrelevant to the cancer process in terms of miRNA action. This cancellation is reflected in the gene *transferred index* computed with Equation 2 which yields a negligible *differential inhibition* score of 0.73 for this gene. Moreover, when using the logistic regression model indicated in Equation 3 to perform a *gene set analysis* of the gene *transferred index*, gene *GPR162* will not support the enrichment of any of the functions in which it is involved.

The cumulative effect of several weaker miRNA differential-expression events can also be appreciated, for instance, in the results produced for the cancer growth regulator gene *GREB1*. This gene is targeted by 16 miRNAs none of which has an absolute *differential inhibition* score higher than 10 in the analysis of the *esophageal carcinoma* (ESCA) dataset. Nevertheless, adding up all 16 values, we computed a *differential inhibition* score of − 53.65 for the gene, indicating strong inhibition in normal samples compared to tumors. We concluded that *GREB1* is usually regulated in normal tissues by the combined action of many miRNAs, and that this regulation is lost in ESCA tumors, which therefore may affect cancer growth. Regarding the *gene set analysis*, *GREB1* will support the GO terms to which it belongs as being inhibited by miRNA action in controls or, equivalently, as deregulated in cases.

### 3.3 Gene set level

Once the miRNA differential-expression evidence is transferred to the genes, the *differential inhibition* ranking index can be easily analyzed in terms of *gene sets* using a logistic regression approach (Montaner and Dopazo, 2010; Montaner *et al.*, 2009; Sartor *et al.*, 2009).

Table 5 shows the number GO terms enriched in positive and negative *transferred index* values. In our analysis, the positive transferred index values belong to genes whose targeting miRNAs are overexpressed in cancer. These genes are generally more inhibited in tumor samples due to the effect of miRNAs. Therefore, GO terms enriched in the positive transferred index gene values represent biological functions which are globally more inhibited, or intercepted, by the miRNA effect in cases than in controls. Similarly, GO terms enriched in negative transferred index gene values represent those which have higher interception rates in control samples than in tumor samples. The biological interpretation of this second group of functions is that ordinarily they are controlled by miRNA action in normal tissue and that this coordination is lost in affected tissue, causing deregulation of the function in a cancer state. Hence, in this paper we refer to the GO terms enriched in positive transferred index values as *inhibited* or *intercepted* in cancer cells, and we term *gene sets* enriched in negative transferred index values *deregulated* in cancer states. Figure 2 outlines and summarizes the key parameters and steps in our methodology.

**Table 5.** Number significant GO terms in the functional profiling analysis for the paired and unpaired comparisons

	Unpaired			Paired		
ID	Derg.	noDif	Inh.	Derg.	noDif	Inh.
BLCA	2	5167	0	2	5167	0
BRCA	3	5166	0	0	5167	2
CESC	0	5169	0	1	5167	1
COAD	18	4930	221			
ESCA	2	5167	0	1	5168	0
HNSC	53	5116	0	0	5169	0
KICH	1	5167	1	30	5138	1
KIRC	0	5159	10	5	5163	1
KIRP	4	5165	0	13	5155	1
LIHC	7	5080	82	0	5169	0
LUAD	0	5169	0	0	5169	0
LUSC	0	5169	0	0	5169	0
PAAD	3	5165	1	0	5169	0
PCPG	0	5169	0	0	5166	3
PRAD	0	5168	1	1	5168	0
READ	0	5157	12			
SKCM	121	5043	5			
STAD	5	5164	0	0	5169	0
THCA	2	5167	0	2	5167	0
UCEC	89	5080	0	9	5160	0

Columns **Inh.** indicates the number of terms with a **positive** *α* coefficient in the logistic regression analysis. Those are the terms inhibited or intercepted in cases. Columns **Derg.** indicates the number of terms with a **negative** *α* value. Those are the terms inhibited in controls or *deregulated* in cases. Columns noDif indicate the number of GOs with a not significant slope coefficient.

Overall, the GO *inhibition* or *deregulation* patterns found in the paired and unpaired analyses are strongly positively correlated (see Supplementary Materials), reflecting the consistency of our approach. Despite this, the number of GO terms enriched in the paired and unpaired analyses differ, which may reflect inter-individual variability in the role that miRNAs play in cancer. No association pattern between GO size (number of genes in the block) and significance levels was found (see Supplementary Materials), indicating the method’s lack of bias in this respect.

Not many enriched GO terms are shared across cancer types (see Supplementary Materials). This is expected due to the great number of differences in the tissues, both normal and tumoral, collected in the different experiments held in *The Cancer Genome Atlas*. But may also reflect the specific roles miRNAs play in cancer development. Most of the enriched terms shared across different cancer types are related to *cell development*, widely known to be related to cancer evolution. On the other hand, the majority of GO terms which are individually enriched in the different specific cancer types are related to cell *development*, *adhesion*, *signaling* and *proliferation*; all of them major processes associated with cancer.

For instance, in our *paired* analysis, the *endoplasmic reticulum lumen* cellular component (GO:0005788) is deregulated in *BLCA*, *CESC* and *UCEC*, all closely related urogenital carcinomas. Full *gene set* profiling of the paired an unpaired datasets for 5169 GO terms can be found in our Supplementary Materials. It includes comparisons between paired and unpaired subsets and a clustering analysis of the different cancer types, based on GSA results.

In order to estimate type 1 errors, all the analyses where repeated after random permutation of the gene column in the miRNA targets database. This re-sampling procedure preserves the number of genes each miRNA targets and the GO annotations, but removes all biological associations within, and between miRNAs. In these permutation experiments the proportion of significant GO terms remained well below the expected 5% (see Supplementary Materials).

Following the (Godard and van Eyll, 2015) paradigm, logistic regression analysis was also carried out directly at the miRNA level. This can trivially be done using the mdgsa library after the annotation is extrapolated from genes to miRNAs. Functional results at the miRNA and gene level showed a significant positive correlation (see Supplementary Materials).

In order to illustrate how the functional profiling can be restricted to just the expressed genes if such information is available, we downloaded gene expression measurements for the **KICH** dataset and repeated the analysis modifying Equation 2 as indicated in the methods section. As expected, a significantly positive but not too strong correlation was found between the results with and without accounting for the expressed genes. Details of the analysis and results are available in the Supplementary Materials.

## 4 Conclusions

We have introduced a novel approach to the functional interpretation of miRNA studies which is primarily designed to unravel the effects of differential miRNA expression on groups of genes or *pathways*.

Our proposal relies on the *gene set analysis* paradigm which extends currently used *over representation* methodologies. It constitutes a general framework applicable in most genomic scenarios, even when no (or too many) miRNAs are differentially expressed, hence, this algorithm eradicates the arbitrariness of current ‘ad hoc’ procedures. But more importantly, our algorithm can encompass biologically relevant events which are neglected by others, representing a step forward in miRNA gene-regulation modeling. First, our approach accounts for *cancellation effects* that arise when a gene is intercepted by different sets of miRNAs within each biological condition. Second, it is able to incorporate the *additive effect* caused when several weak miRNA inhibitors exert their influence on the same gene.

These major advantages are possible thanks to a key innovative idea introduced in this paper: that differential miRNA expression can be meaningfully *transferred* to the gene level as a *differential inhibition* score.

If miRNA-to-gene transfer comprises cancellation and summation effects, the *gene set* methodology performs the same role at the functional level. A GO term is considered not to be enriched, or *canceled*, if half of its genes are inhibited in cases and the other half in controls. But also the *additive effect* consideration reappears at pathway level: many weakly deregulated, or inhibited, genes which would be inconsequential in isolation become relevant if they are systematically annotated under the same biological function.

Besides the analysis presented here, the logistic regression methodology developed in our previous work allows the algorithm to be extended in many convenient ways. For example, the relative importance of miRNAs, genes, or the miRNA-gene relationship can be easily weighted for at the *transference* step or when fitting the logistic model. Thus, confidence about the miRNA targets, number of target sites in genes, absolute gene expression levels, or even natural miRNA functional loss (Carbonell *et al.*, 2012), can be directly accounted for using our model. Furthermore, additional genomic information can be incorporated using our multidimensional framework: for instance, joint GSA analysis of miRNA regulation and gene expression is straightforward once the *transference* problem is solved using the methodology we explain in this paper. Also the flexibility of our approach and software makes its use independent of the differential-expression algorithm used at the miRNA level. Different statistical tests or even fold changes can substitute the edgeR method used here; similarly, any miRNA target databases can be used.

We have illustrated our novel methodology using an extensive collection of cancer datasets, but here we just present some deregulated genes or functions as a proof of concept. Complete results are available in the supplementary data. We hope that the ideas introduced here can easily be extrapolated to other gene regulatory processes such as those involving transcription factors for instance.

Finally, it is crucial to highlight the importance of data normalization for the correct functional interpretation of NGS studies. Inadequate data preprocessing may affect *P*-values for differential miRNA expression and even the sign of test statistics, sequentially affecting Equations 1–3 and therefore changing the results of our methodology. Thus, thorough data preparation and exploration should always be conducted before using our algorithm.

## Supplementary Material

Supplementary Data
