# Supplementary material for: Integrated gene set analysis for microRNA studies
Source: Bioinformatics. 2016 Jun 20;32(18):2809–16. doi: 10.1093/bioinformatics/btw334 (PMC5018374; doi:10.1093/bioinformatics/btw334)
Supplement: Supplementary Data [file supp_32_18_2809__index.html]

Integrated gene set analysis for microRNA studies — Integrated gene set analysis for microRNA studies — Supplementary Data 

# Integrated gene set analysis for microRNA studies

## Supplementary Data

files

- Supplementary Data - zip file
