# Supplementary material for: Integrated gene set analysis for microRNA studies
Source: Bioinformatics. 2016 Jun 20;32(18):2809–16. doi: 10.1093/bioinformatics/btw334 (PMC5018374; doi:10.1093/bioinformatics/btw334)
Supplement: Supplementary Data [file supp_btw334_suppl_data.zip › godard_comparison_report.pdf]

Integrated Gene Set Analysis for microRNA Studies

Comparison of methods for Gene Set Analysis

December 22, 2015

Contents

1 Methods **2**

2 Results **3**

1 Methods

A GSA extension of Godard's approach have been computed straight forward using logistic regression models and our Bioconductor library. This strategy will certainly retain Godard's methodology good characteristics while incorporating the benefits of the GSA approach over the ORA one.

We did find that the functional results at miRNA level (Godard's generalization) and the ones at gene level (after transference as originally proposed) have a significantly positive correlation. This indicates that overall both methodologies should provide similar findings. The correlation is not very strong though as the methodologies are explicitly different.

Detailed results and scripts are available in `https://github.com/dmontaner-papers/gsa4mirna` (folders: supplementary_files_godard and scripts_godard).

2 Results

For each cancer type, there are several plots displaying the correlation between the GSA analysis carried out at miRNA level (Godard's paradigm) and at gene level after "transference". Each dot represents a GO term. X and Y values are derived from p-values and signs of the log odds ratios resulting from the mdgsa analysis (similar to equation 1 of the paper but at GO level instead of at miRNA level).

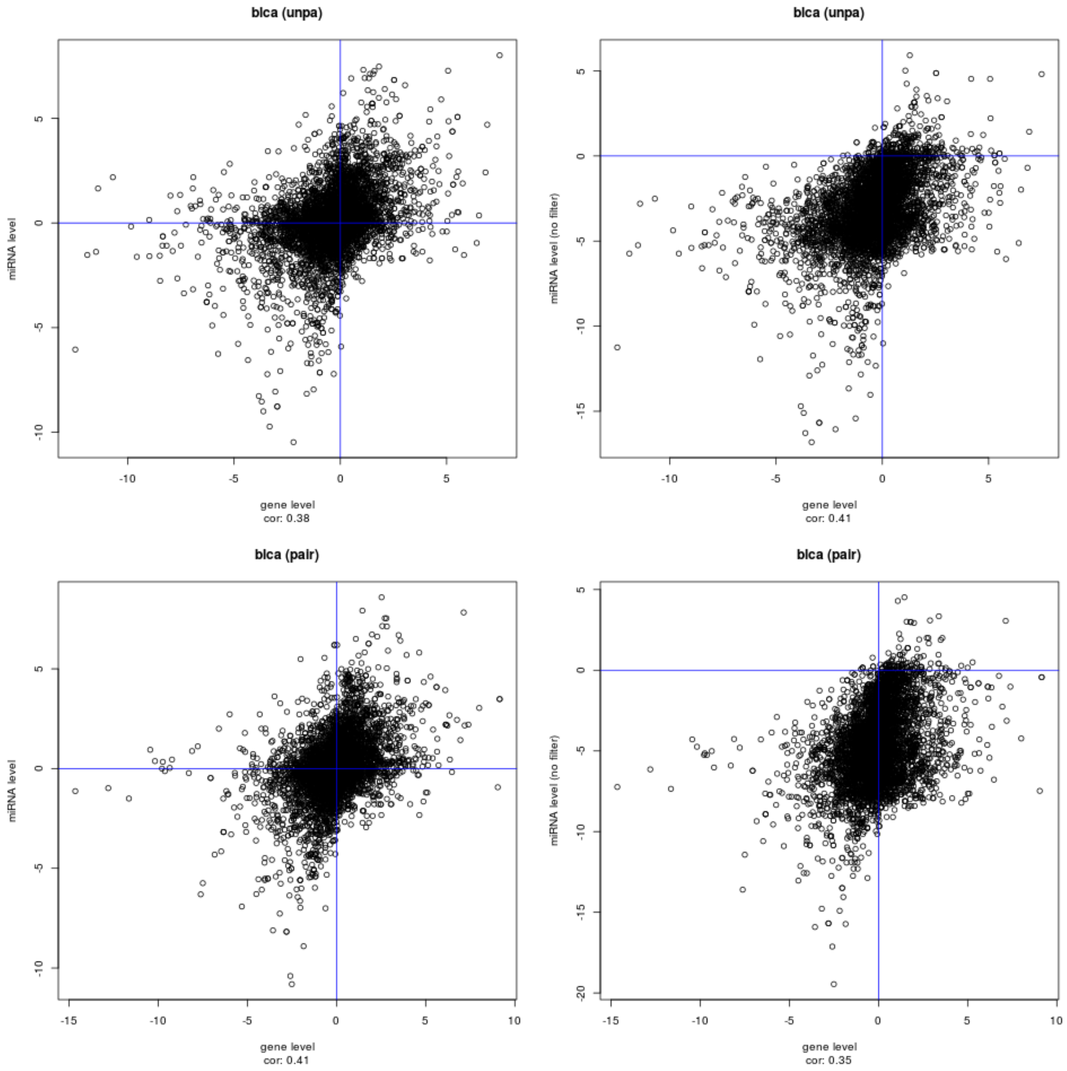

Figure 1: BLCA

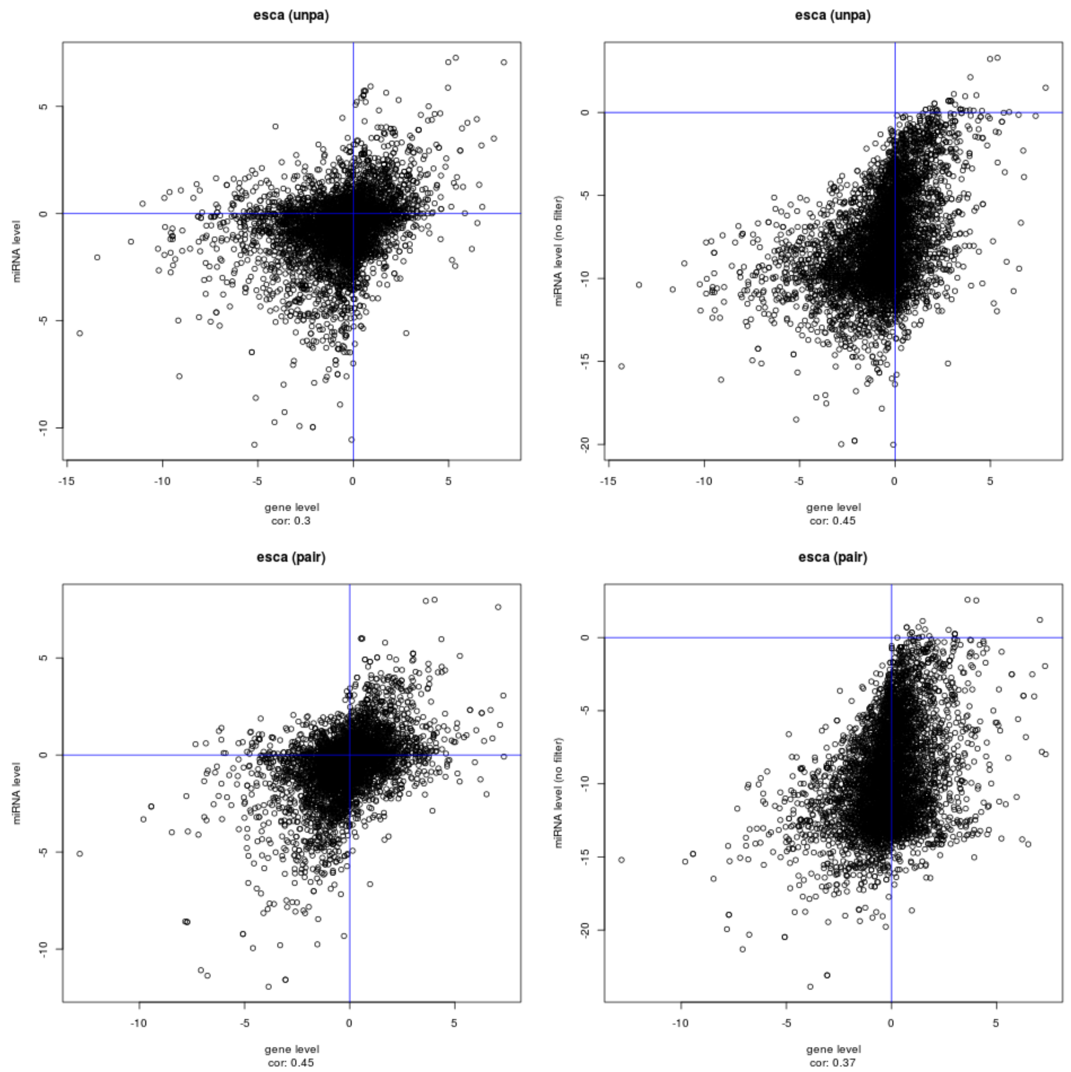

Figure 2: ESCA

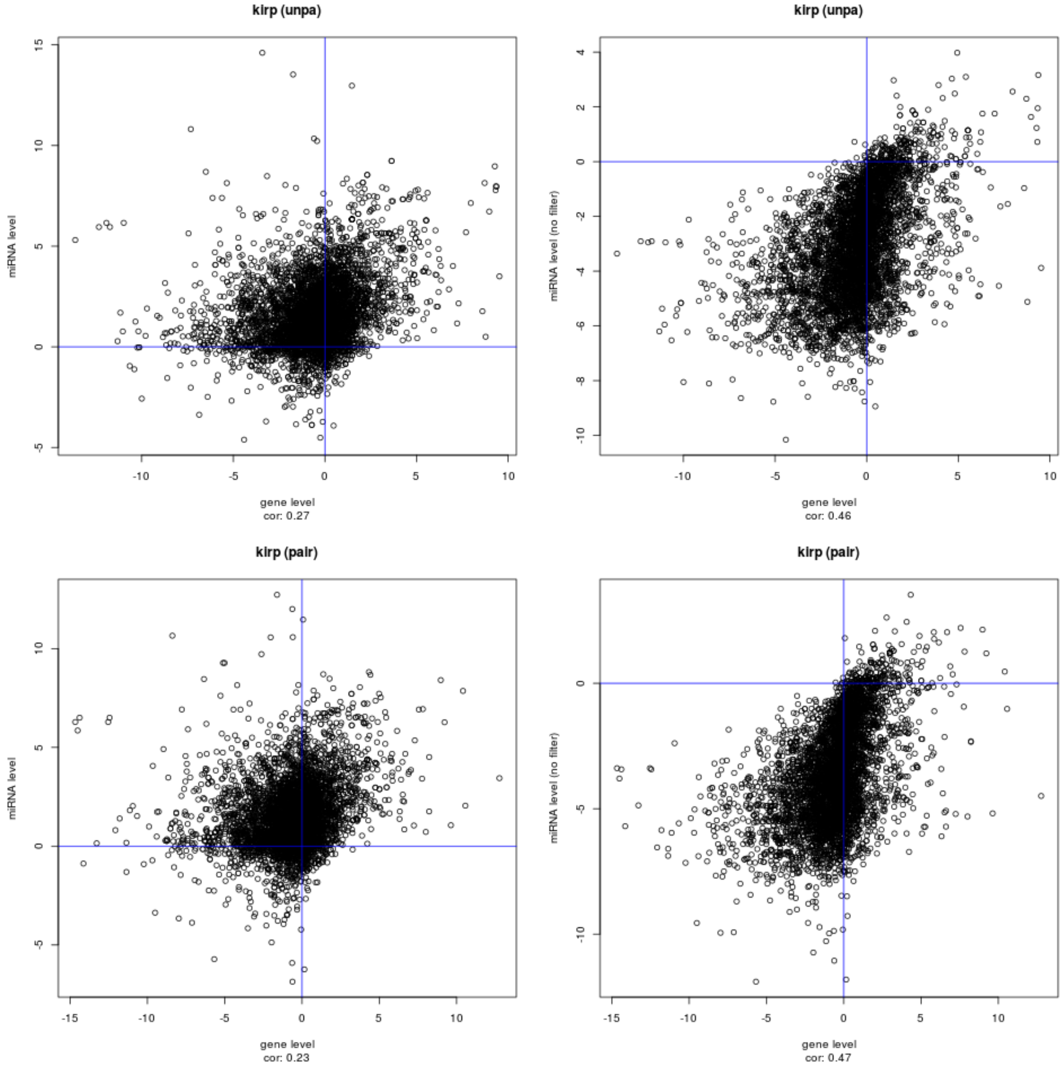

Figure 3: KIRP

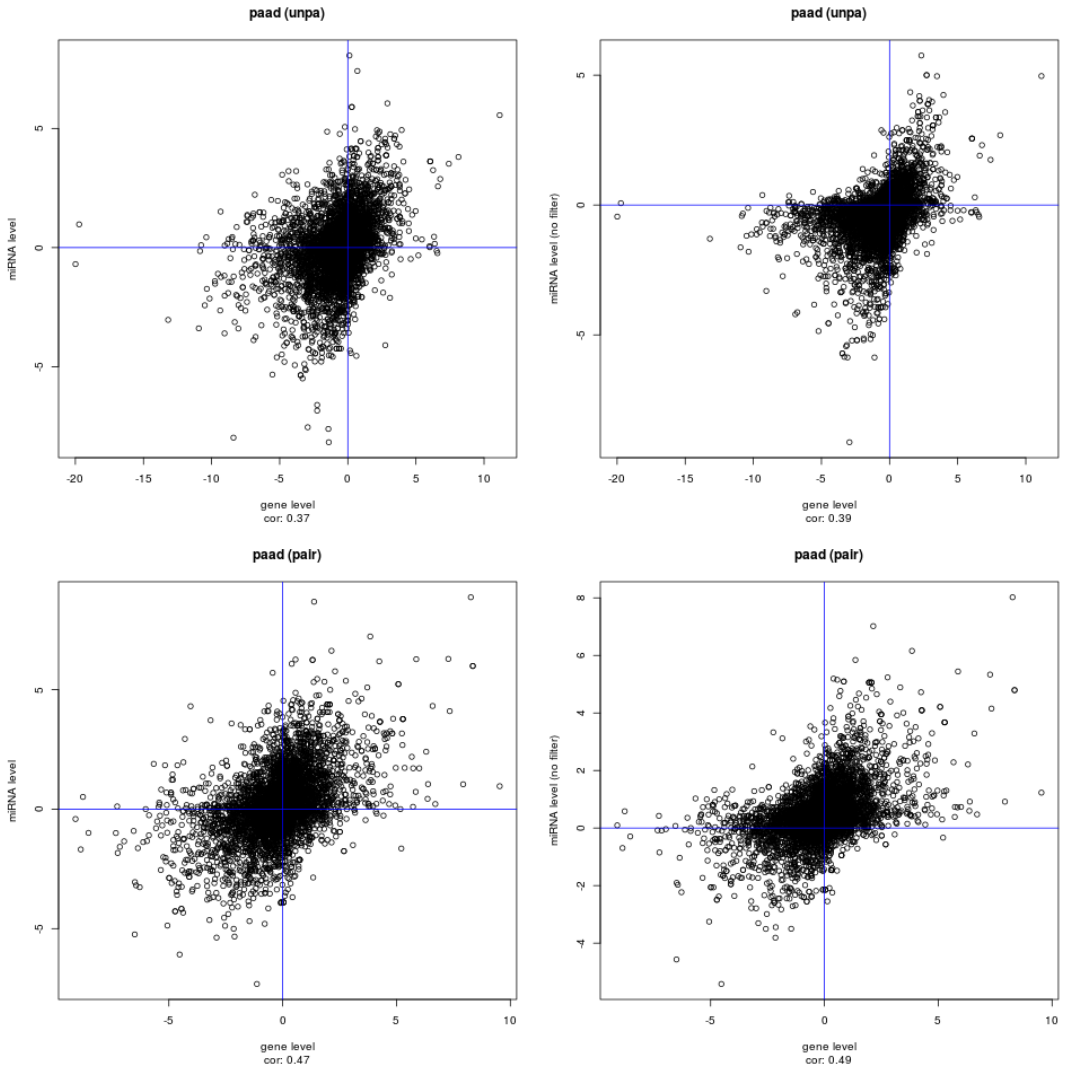

Figure 4: PAAD

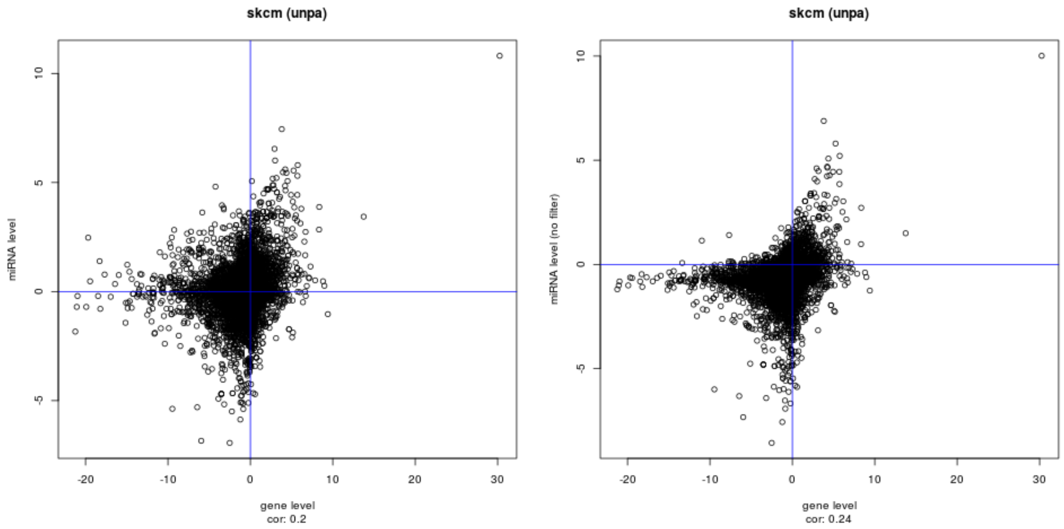

Figure 5: SKCM

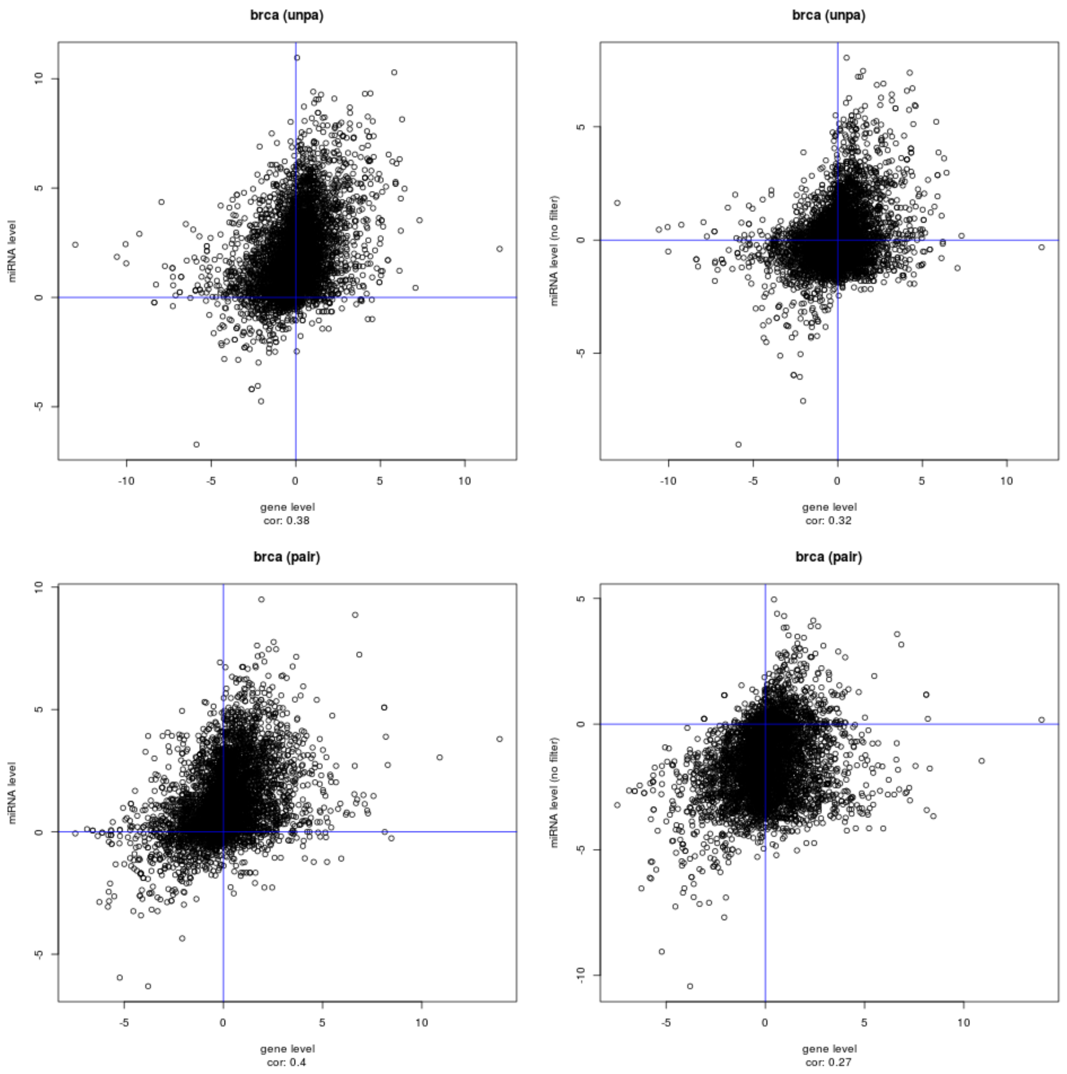

Figure 6: BRCA

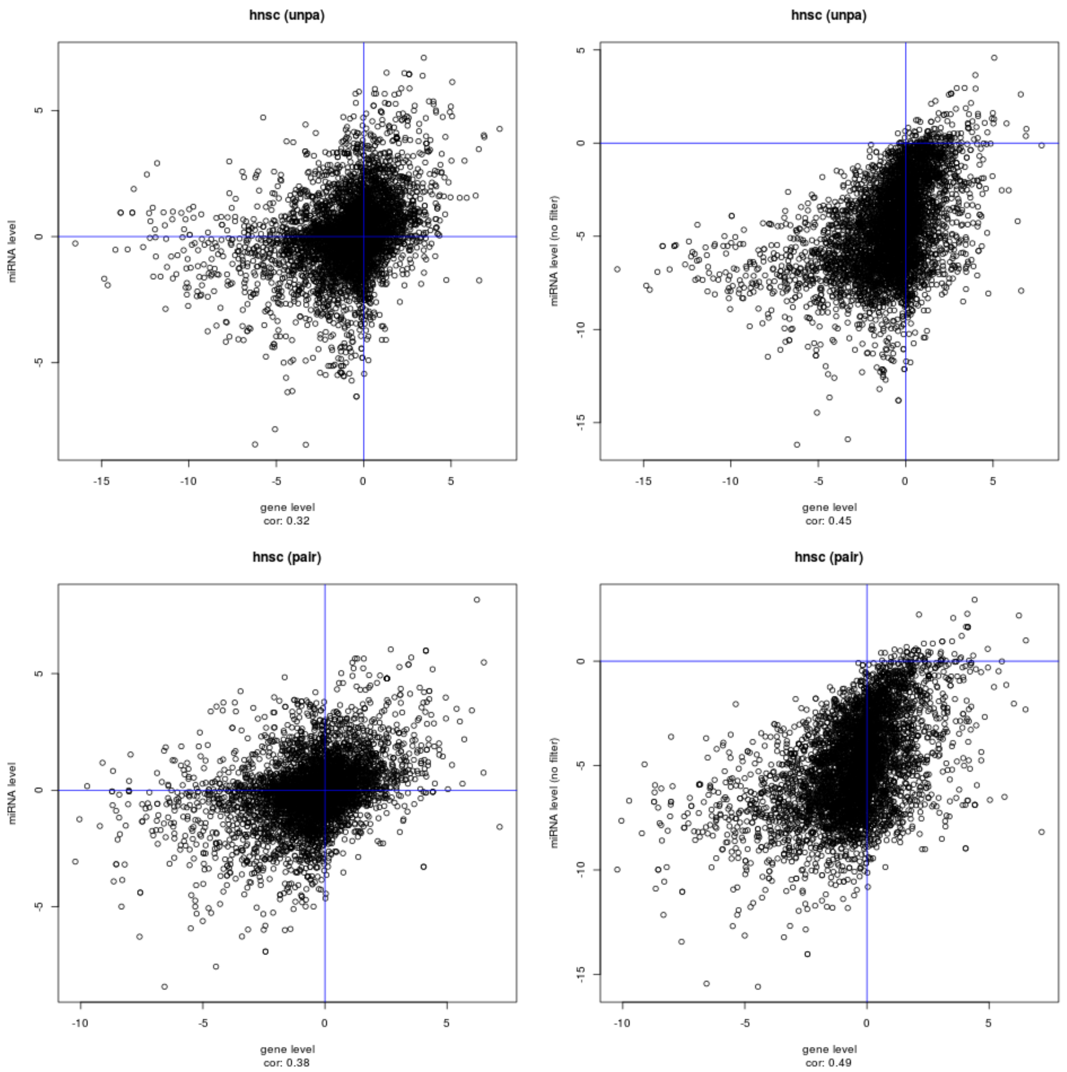

Figure 7: HNSC

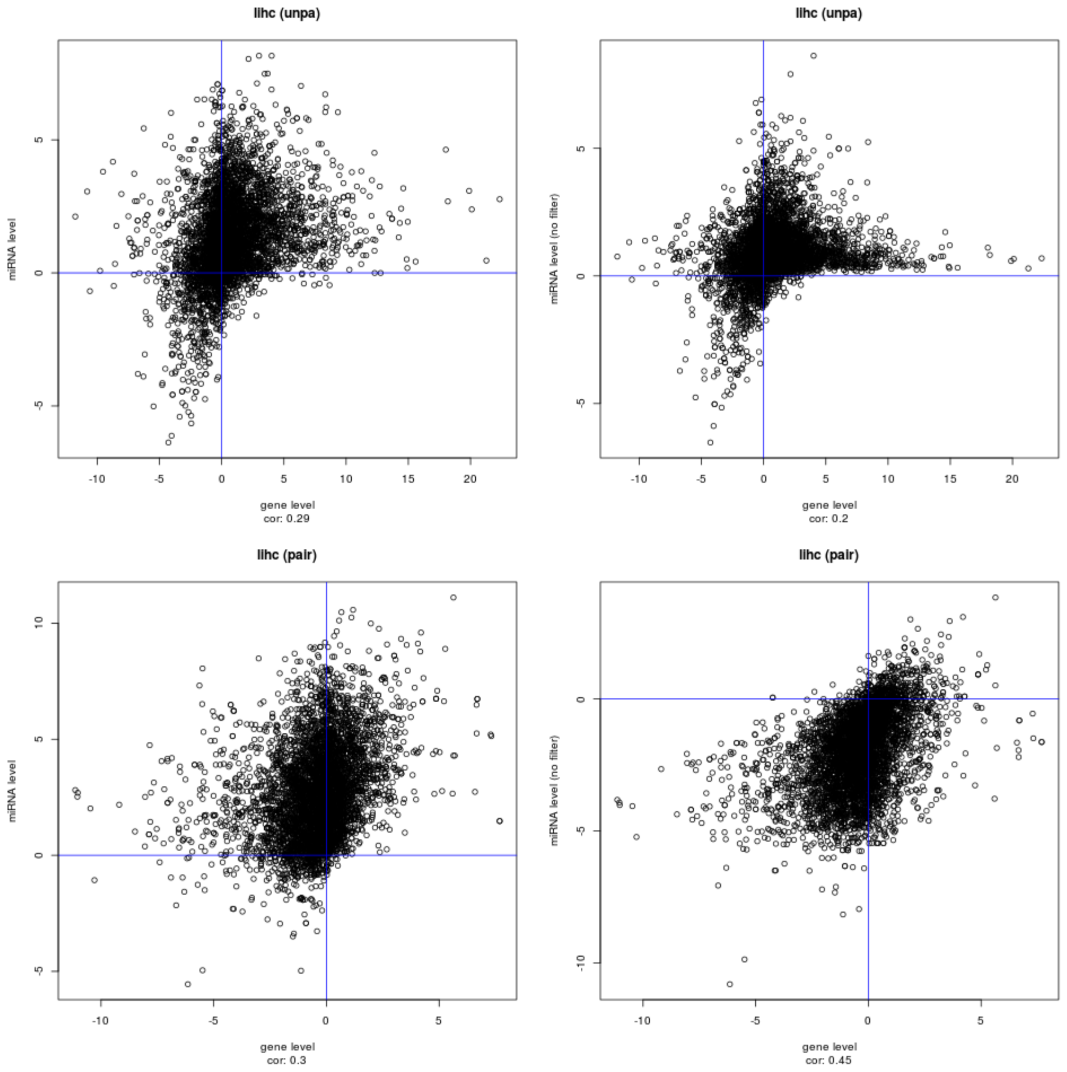

Figure 8: LIHC

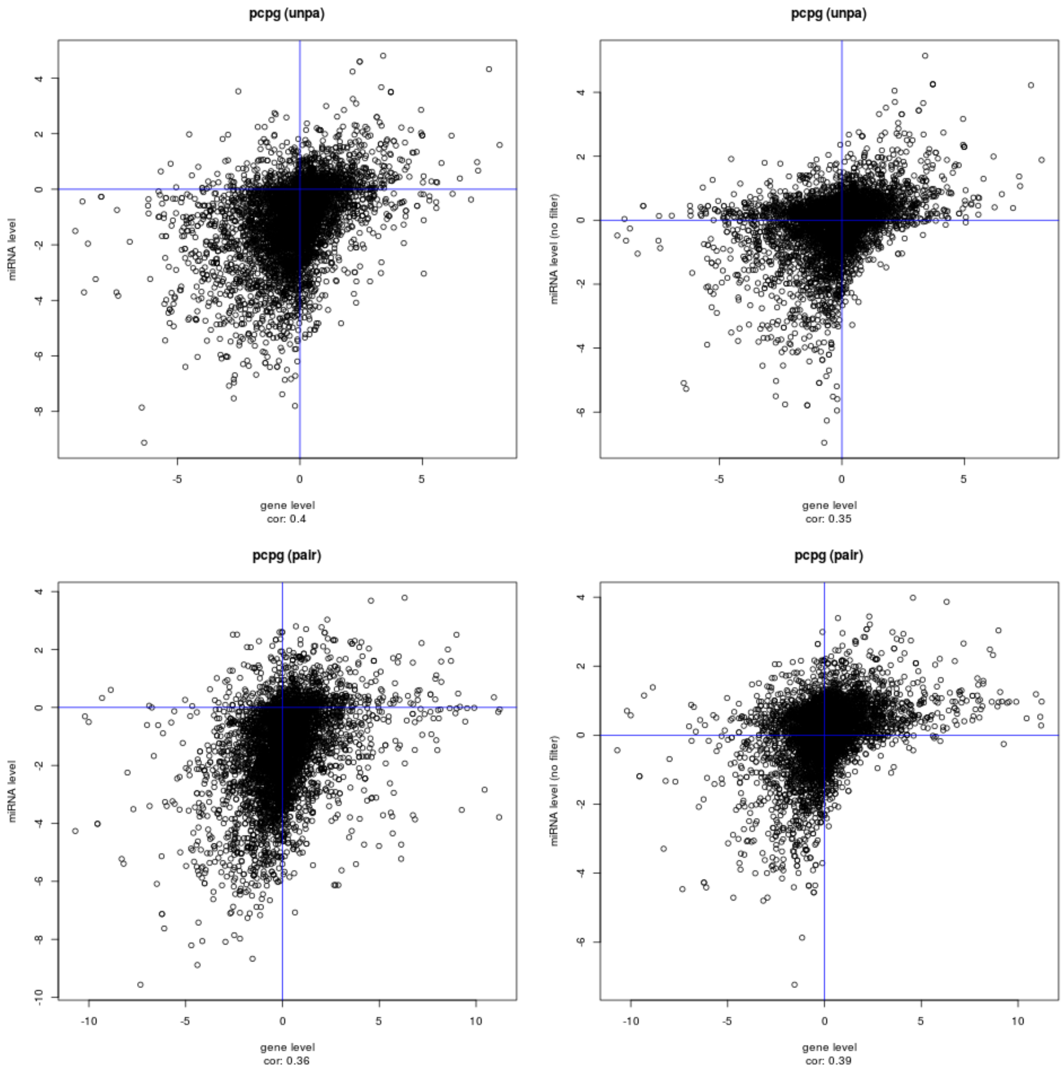

Figure 9: PCPG

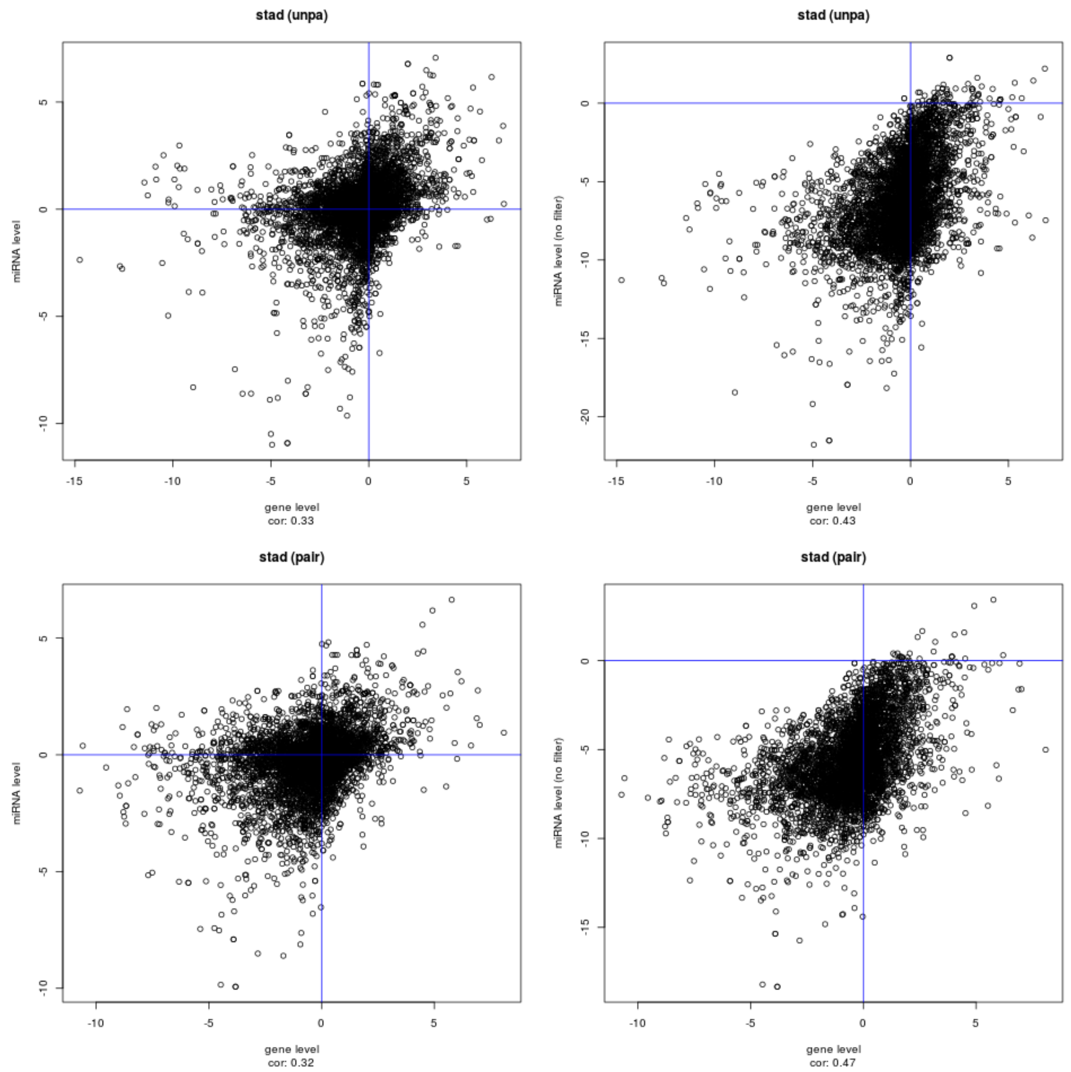

Figure 10: STAD

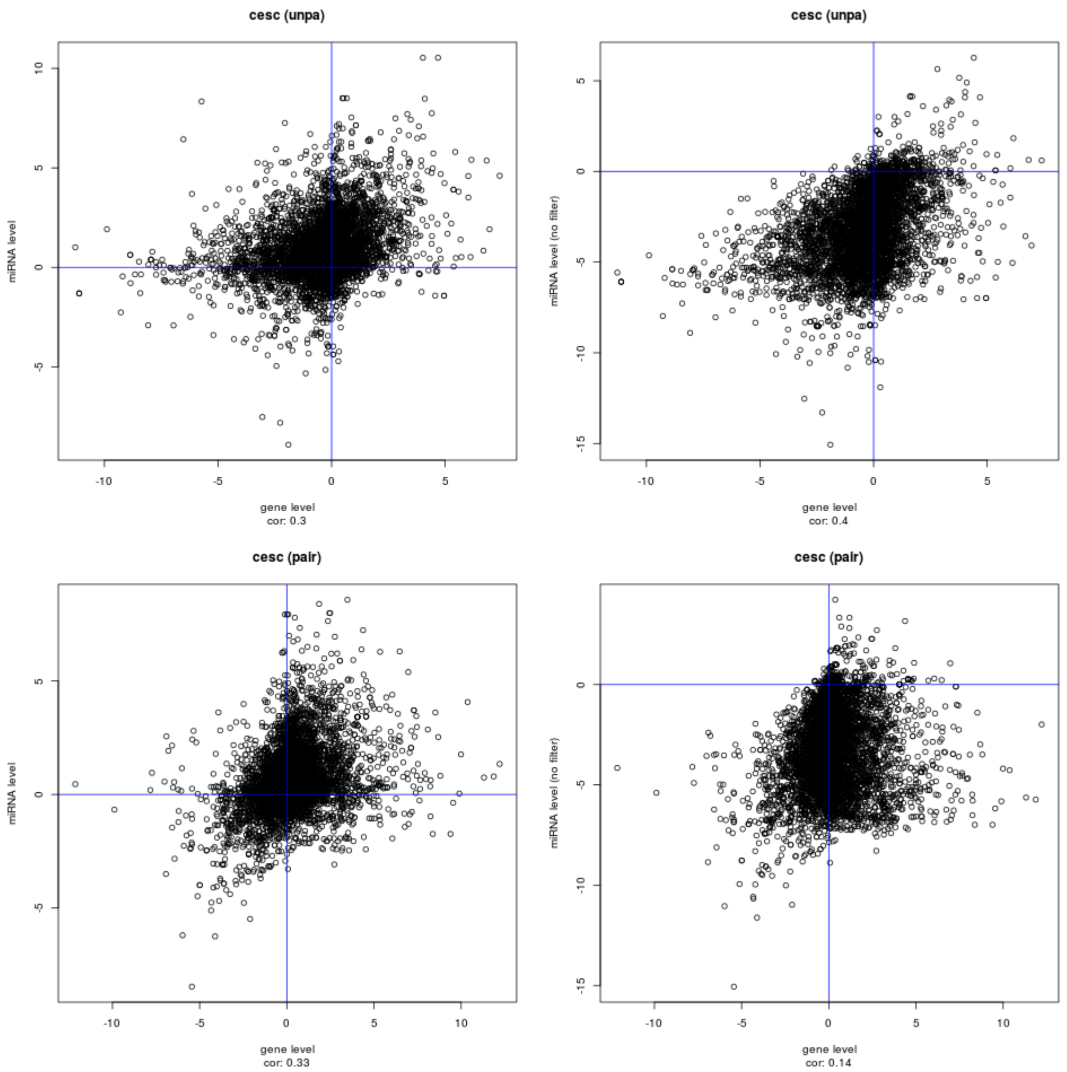

Figure 11: CESC

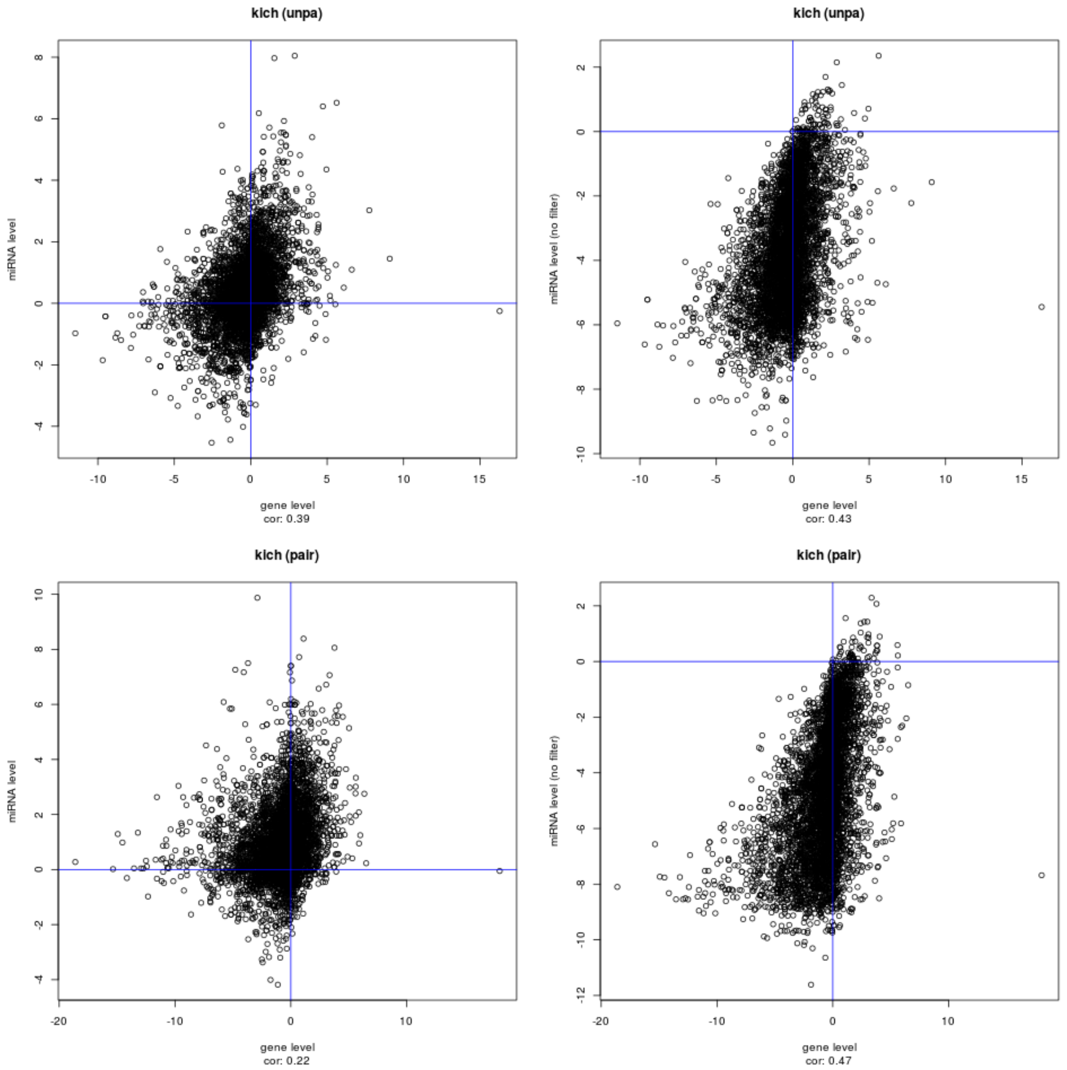

Figure 12: KICH

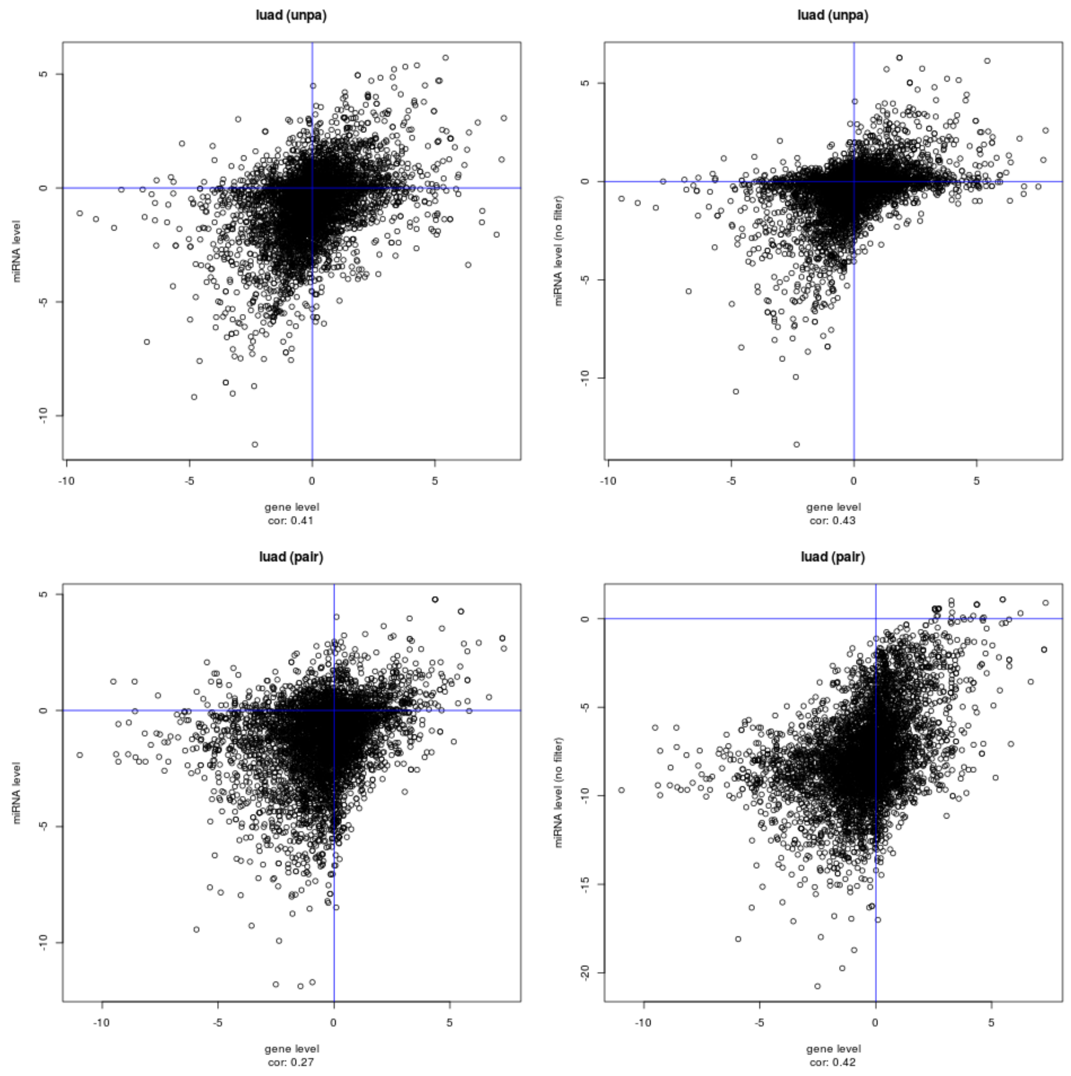

Figure 13: LUAD

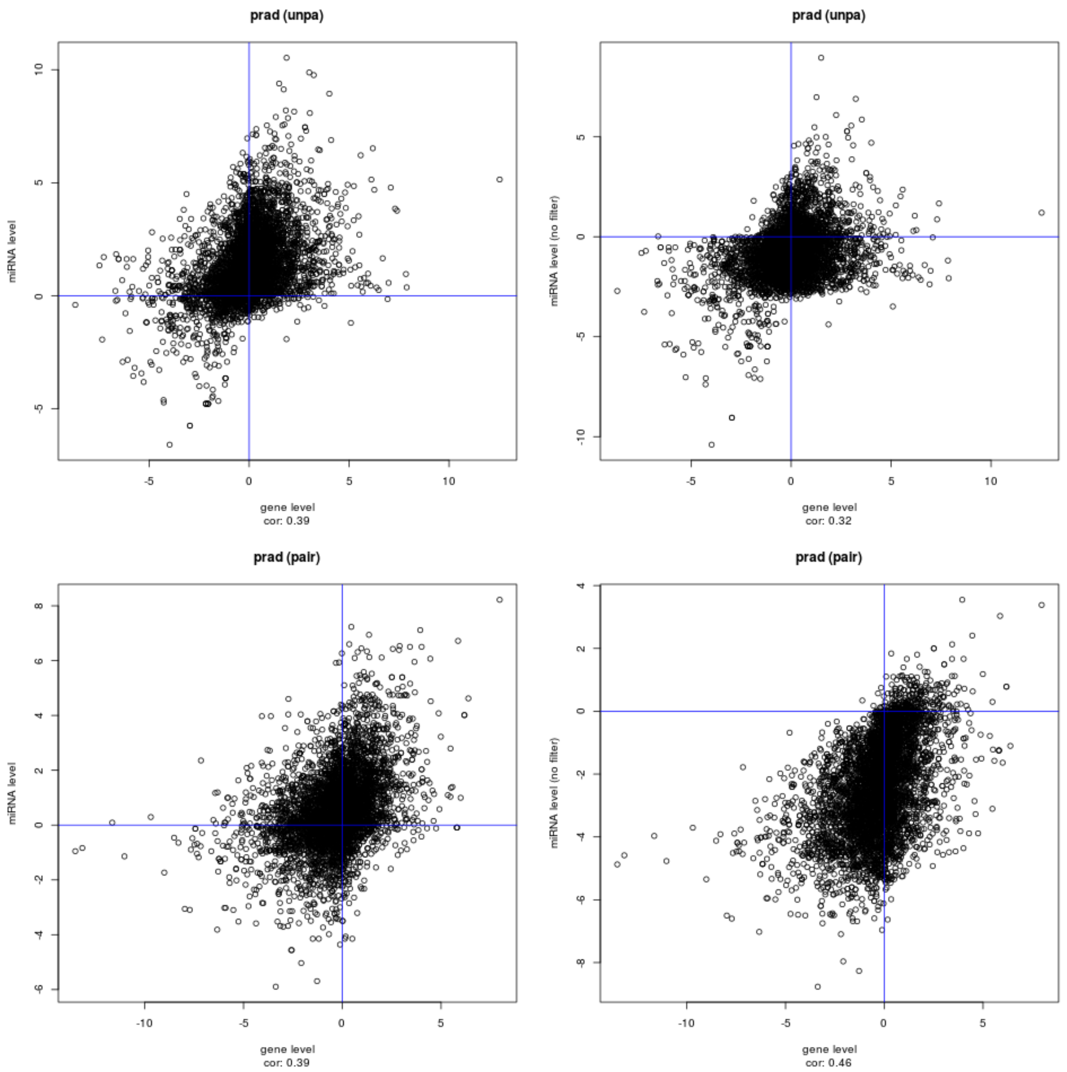

Figure 14: PRAD

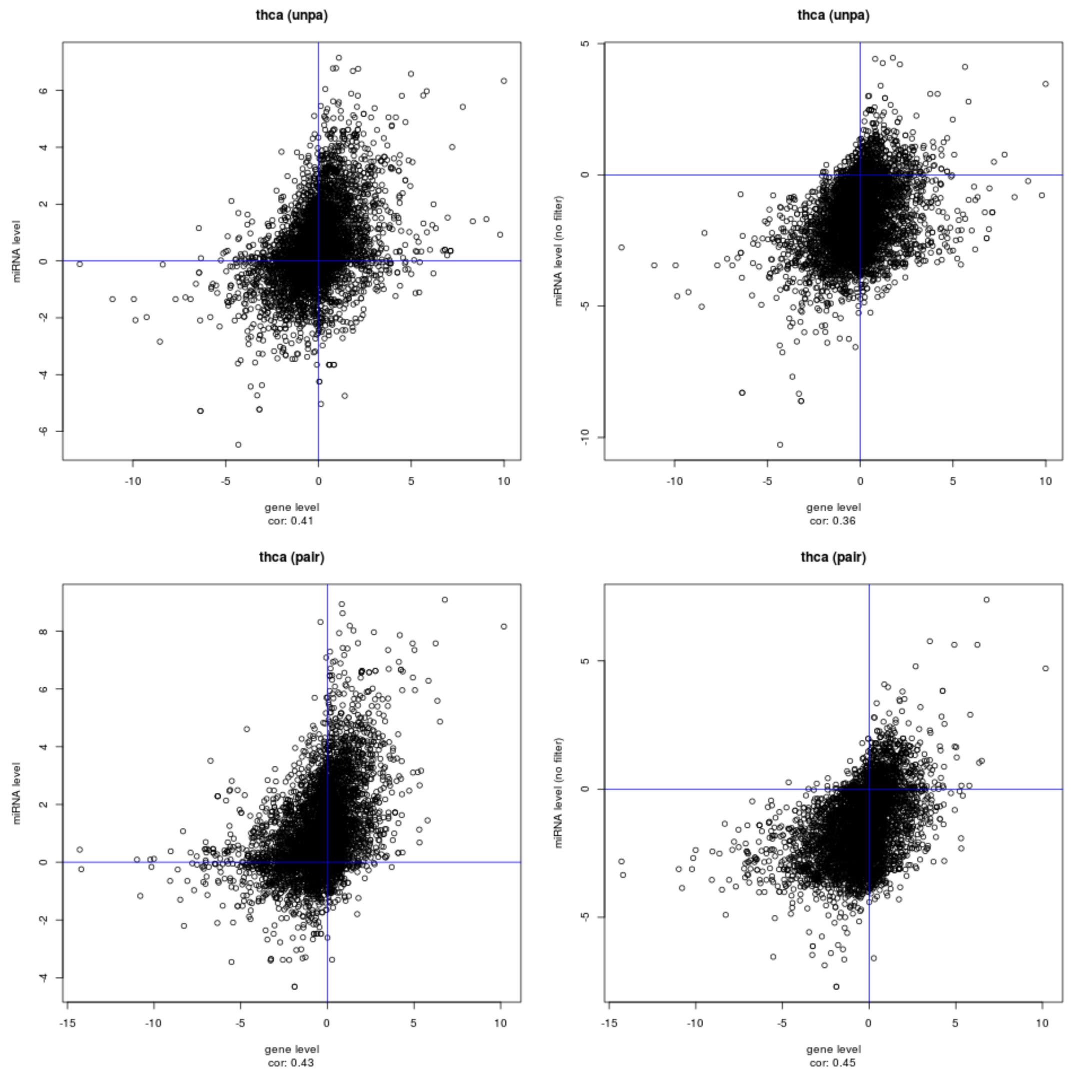

Figure 15: THCA

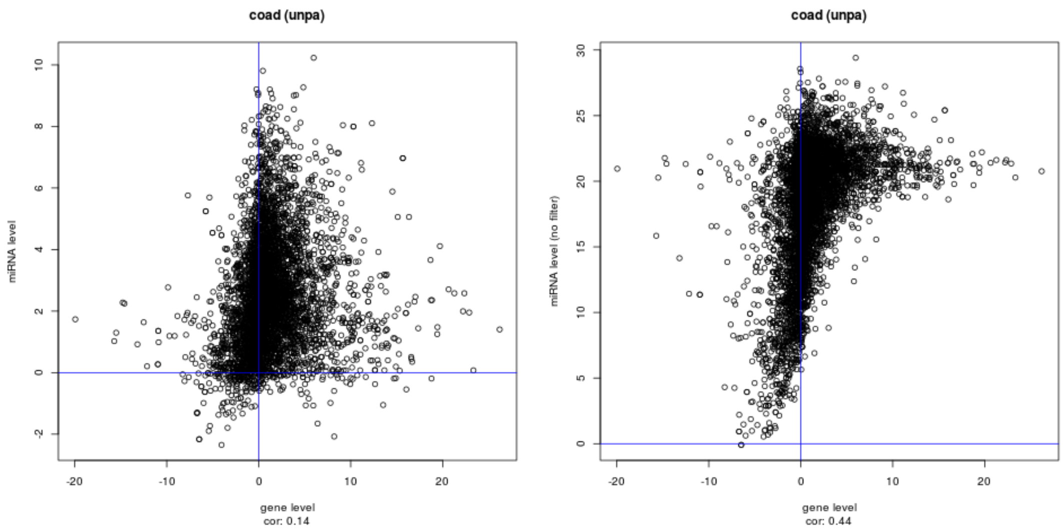

Figure 16: COAD

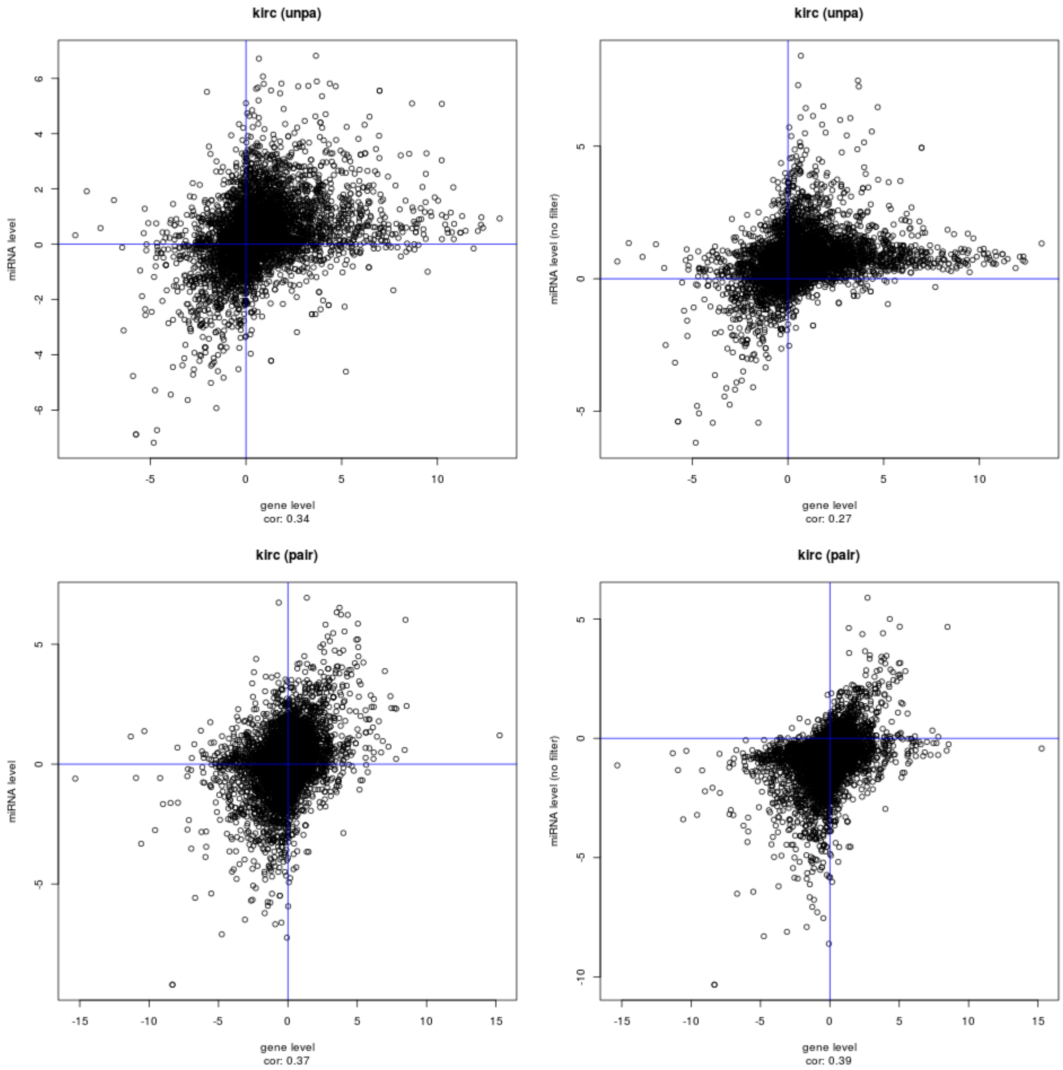

Figure 17: KIRC

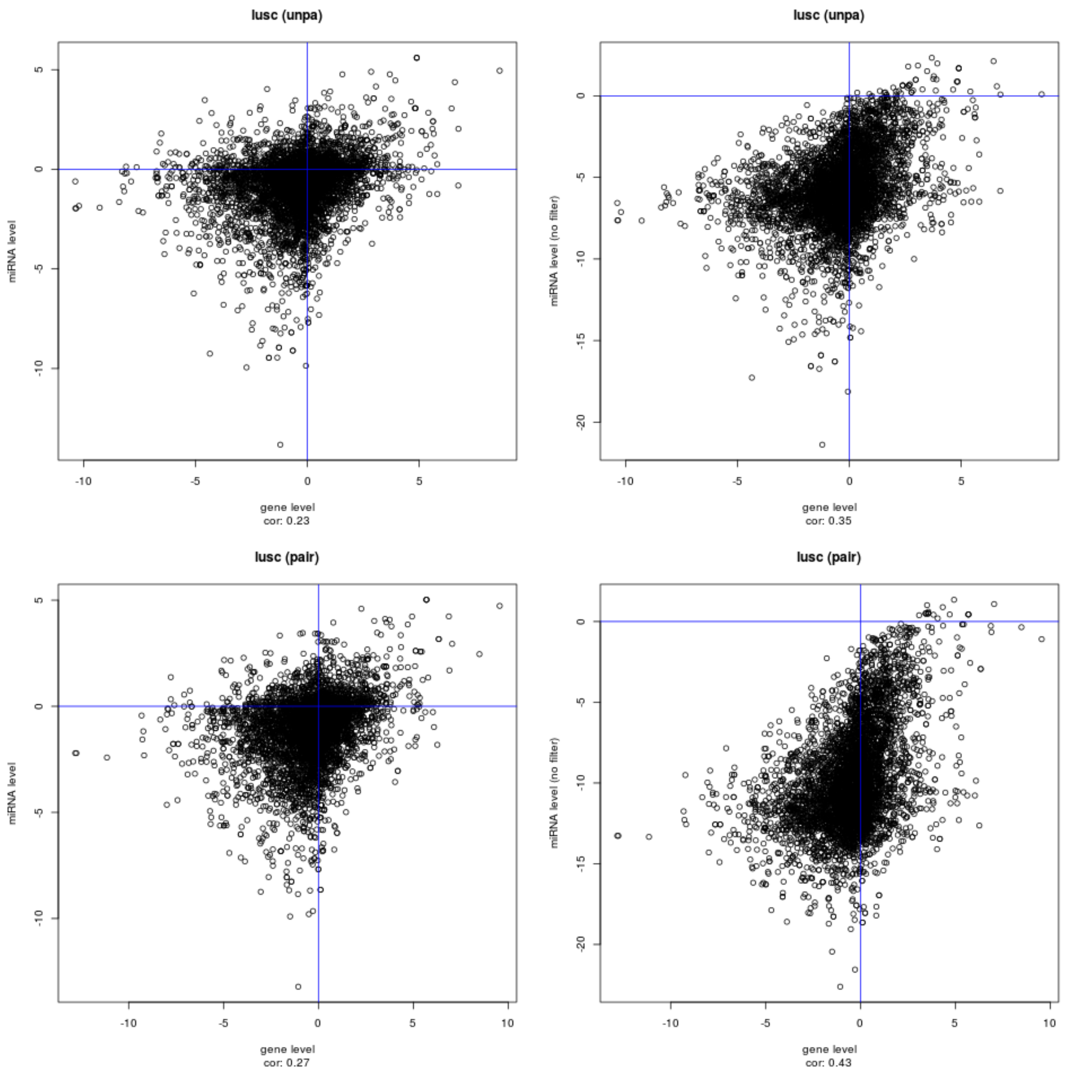

Figure 18: LUSC

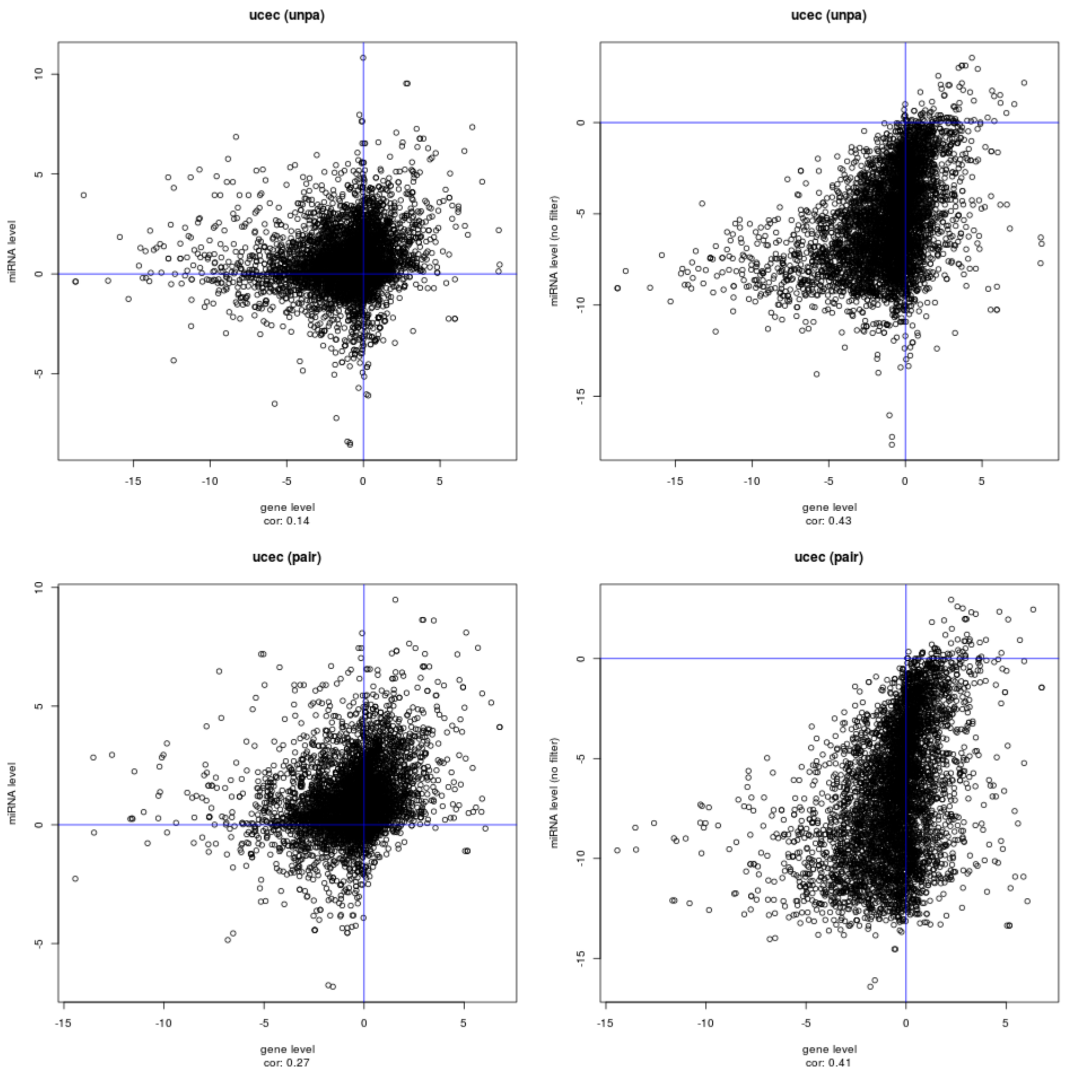

Figure 19: UCEC

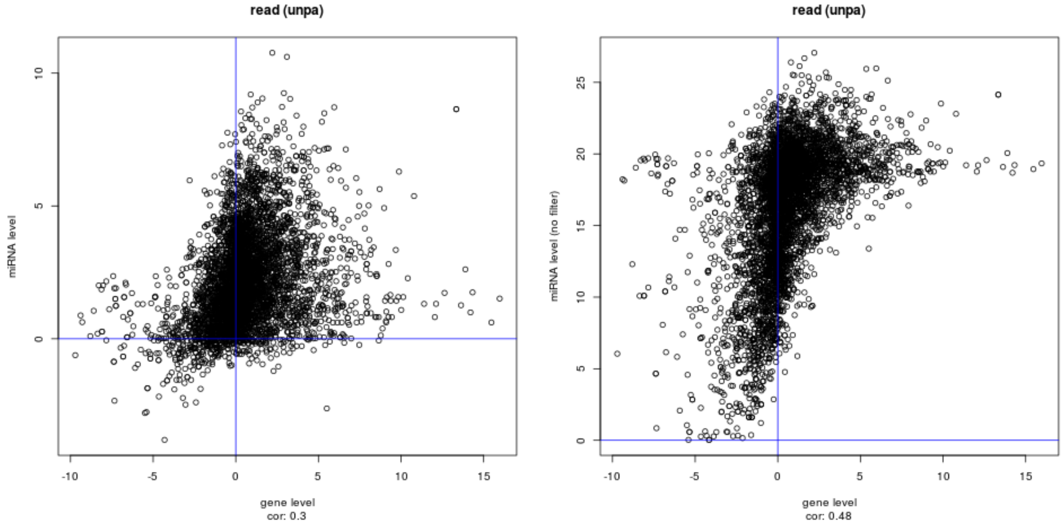

Figure 20: READ
