# Supplementary material for: Integrated gene set analysis for microRNA studies
Source: Bioinformatics. 2016 Jun 20;32(18):2809–16. doi: 10.1093/bioinformatics/btw334 (PMC5018374; doi:10.1093/bioinformatics/btw334)
Supplement: Supplementary Data [file supp_btw334_suppl_data.zip › permutation_analysis_report.pdf]

Integrated Gene Set Analysis for microRNA Studies

Estimation of the type I error rate

2016-02-05

Methods

The estimation of the type I error rate have been computed from a permutation strategy of the data.

We have created a table of all miRNA - gene target pairs, and then randomly permuted the gene column. This would have the effect of preserving the number of genes each miRNA targets, and the genes and the number of miRNAs each gene is associated with, but would remove all biological association within and between miRNAs.

This permutation strategy was performed 100 times. Median and percentiles were used to describe the percentage of significant results.

All scripts are available in https://github.com/dmontaner-papers/gsa4mirna (folder: scripts_permutation).

Results

For each cancer type, this table shows the percentage of significant results.

The amount of False Positives (FP) found in the permuted analysis in **paired studies** remains under the expected thresholds according to the p-value cutoff selected.

Table 1: % FP in paired studies

Cancer	Median	Percentile 5	Percentile 95
BLCA	0	0	0.037
BRCA	0	0	0.055
CESC	0.037	0	0.405
ESCA	0.018	0	0.018
HNSC	0.11	0.037	0.883
KICH	0.258	0.055	0.662
KIRC	0	0	0.055
KIRP	1.031	0.368	2.024
LIHC	0	0	0.147
LUAD	1.104	0.276	1.987
LUSC	1.288	0.57	2.319
PAAD	0	0	0.018
PCPG	0	0	0.055
PRAD	0.147	0	0.865
STAD	0.092	0	0.736
THCA	0	0	0
UCEC	0.129	0	0.645

The amount of False Positives found in the permuted analysis in **unpaired studies** remains under the expected thresholds according to the p-value cutoff selected.

Table 2: % FP in unpaired studies

Cancer	Median	Percentile 5	Percentile 95
BLCA	0	0	0.037
BRCA	0	0	0
CESC	0.202	0.018	0.386
COAD	4.803	3.607	5.332
ESCA	0.755	0.35	1.104
HNSC	0.681	0	2.3
KICH	0	0	0.037
KIRC	0.202	0.037	1.822
KIRP	0.883	0.294	1.307
LIHC	4.141	3.257	6.294
LUAD	0	0	0
LUSC	0.883	0.074	2.079
PAAD	0.018	0	0.129
PCPG	0.018	0	0.055
PRAD	0	0	0.037
READ	0.294	0.018	0.626
SKCM	1.619	0.773	2.687
STAD	0.212	0.018	0.883
THCA	0	0	0.055
UCEC	2.843	1.012	4.734
