# Supplementary material for: Integrated gene set analysis for microRNA studies
Source: Bioinformatics. 2016 Jun 20;32(18):2809–16. doi: 10.1093/bioinformatics/btw334 (PMC5018374; doi:10.1093/bioinformatics/btw334)
Supplement: Supplementary Data [file supp_btw334_suppl_data.zip › clustering_report.pdf]

Integrated Gene Set Analysis for microRNA Studies

Exploratory Analysis for Enrichment Results

August 28, 2015

Contents

1 Overview **2**

2 Principal Component Analysis **3**

3 Clustering Analysis **6**

3.1 Clustering Analysis, euclidean distance 6

3.2 Clustering Analysis, correlation distance 10

3.3 Significant Clustering Analysis, correlation distance 14

References **18**

1 Overview

The goal is to detect similar cancer groups from functional enrichment results. We generate an indicator for each GO term: sign (log(OR)) * -1 * log(raw pvalue) from **mdgsa** (`http://www.bioconductor.org/packages/release/bioc/html/mdgsa.html`). We perform clustering and principal components analysis to explore relationships between groups of tumors.

	ID	total	cases	controls	paired	description
blca	BLCA	271	252	19	19	Bladder Urothelial Carcinoma
brca	BRCA	807	720	87	86	Breast invasive carcinoma
cesc	CESC	218	215	3	3	Cervical squamous cell carcinoma
coad	COAD	243	235	8	0	Colon adenocarcinoma
esca	ESCA	113	102	11	11	Esophageal carcinoma
hnsc	HNSC	519	475	44	43	Head and Neck squamous cell carcinoma
kich	KICH	91	66	25	25	Kidney Chromophobe
kirc	KIRC	311	240	71	68	Kidney renal clear cell carcinoma
kirp	KIRP	245	211	34	34	Kidney renal papillary cell carcinoma
lihc	LIHC	283	233	50	49	Liver hepatocellular carcinoma
luad	LUAD	474	428	46	39	Lung adenocarcinoma
lusc	LUSC	376	331	45	45	Lung squamous cell carcinoma
paad	PAAD	100	96	4	4	Pancreatic adenocarcinoma
pcpg	PCPG	182	179	3	3	Pheochromocytoma and Paraganglioma
prad	PRAD	117	100	17	17	Prostate adenocarcinoma
read	READ	93	90	3	0	Rectum adenocarcinoma
skcm	SKCM	75	74	1	0	Skin Cutaneous Melanoma
stad	STAD	345	306	39	39	Stomach adenocarcinoma
thca	THCA	558	499	59	59	Thyroid carcinoma
ucec	UCEC	418	386	32	19	Uterine Corpus Endometrial Carcinoma

Figure 1: Analyzed datasets. Columns on the table display: TCGA disease ID, the total number of samples in the analysis, the number of tumoral samples, the number of control samples (solid normal tissue), the number of paired samples available in the dataset and the cancer type

2 Principal Component Analysis

The PCA plots below show the first three principal components of all samples in the study. See `http://en.wikipedia.org/wiki/Principal_component_analysis` for details on Principal Component Analysis.

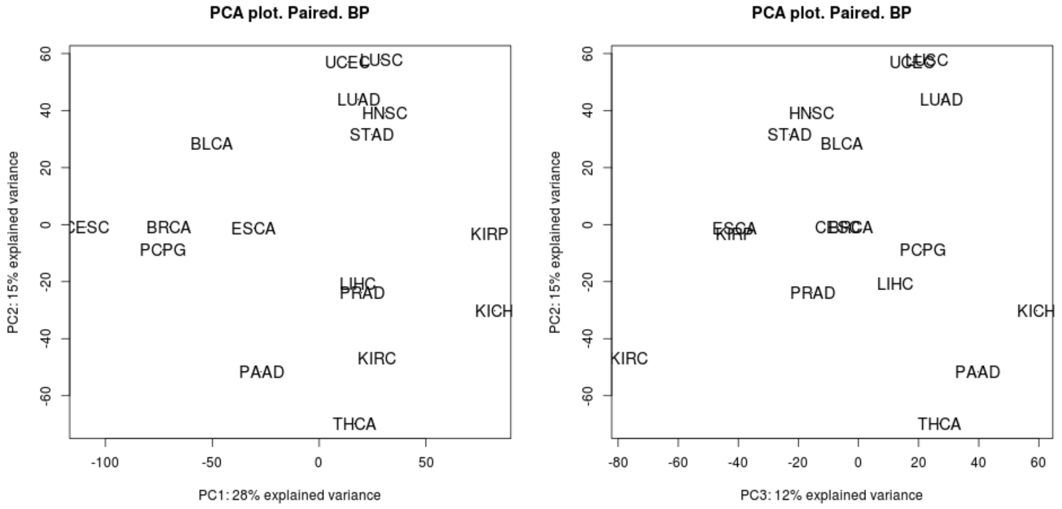

Figure 2: PCAplot from enrichment results (Biological Processes) in TCGA paired studies

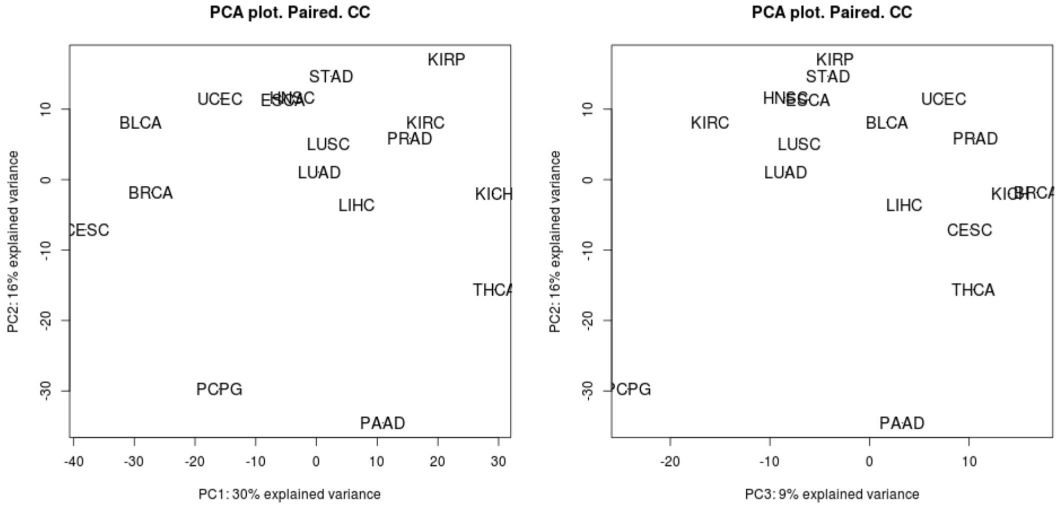

Figure 3: PCAplot from enrichment results (Cellular Components) in TCGA paired studies

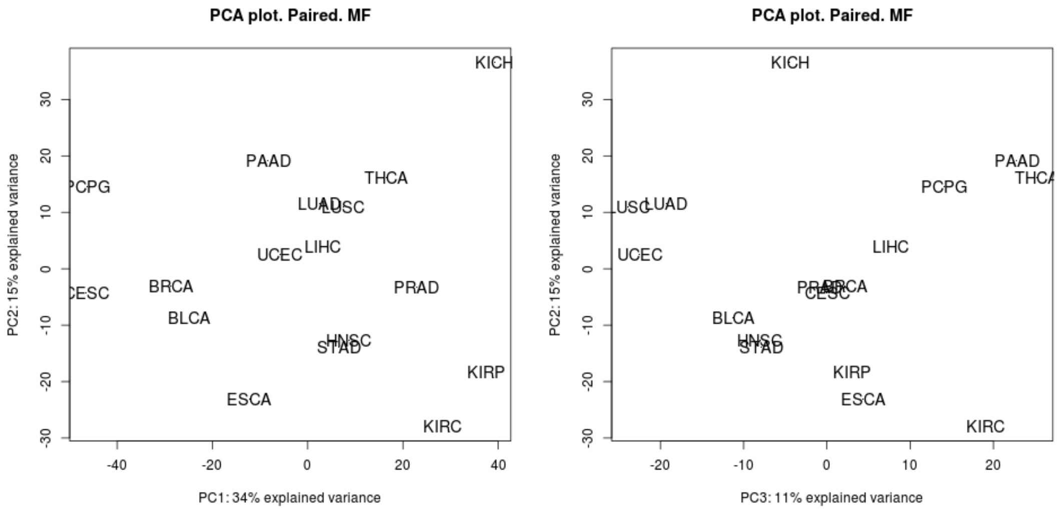

Figure 4: PCAplot from enrichment results (Molecular Functions) in TCGA paired studies

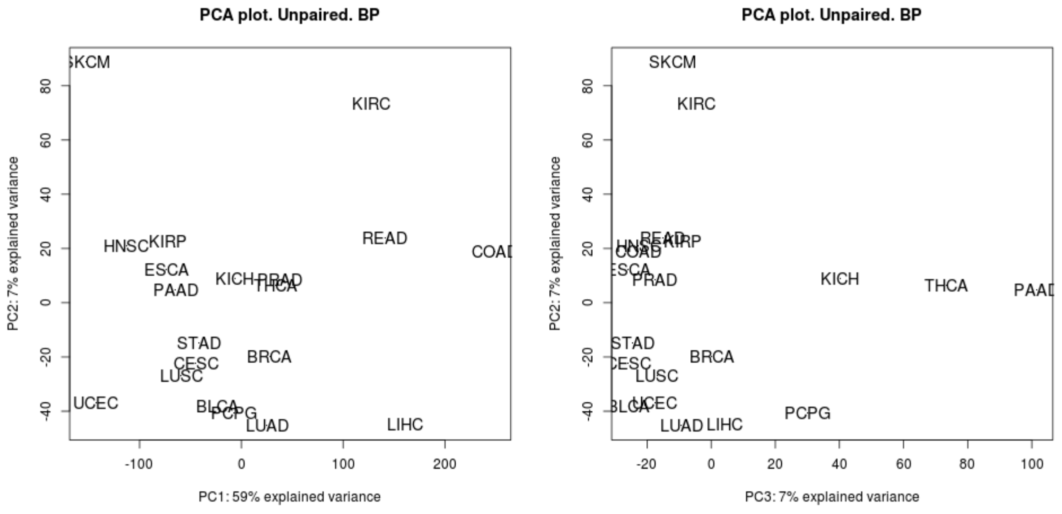

Figure 5: PCAplot from enrichment results (Biological Processes) in TCGA unpaired studies

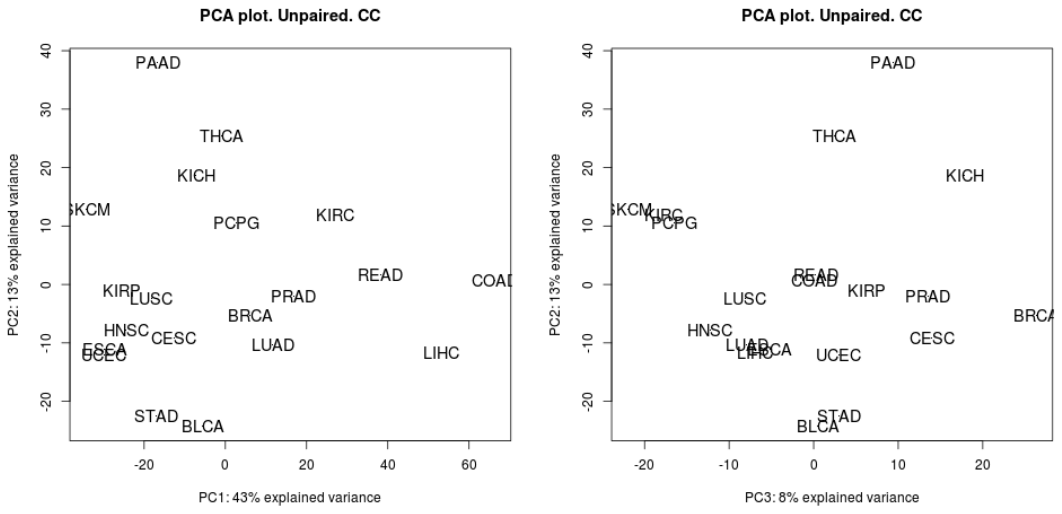

Figure 6: PCAplot from enrichment results (Cellular Components) in TCGA unpaired studies

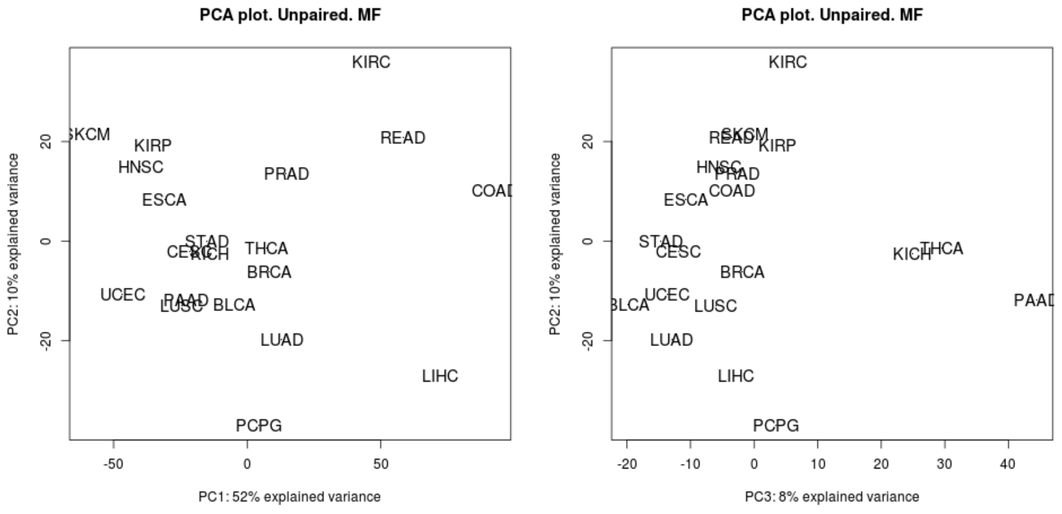

Figure 7: PCAplot from enrichment results (Molecular Functions) in TCGA unpaired studies

3 Clustering Analysis

Complete linkage method was used for hierarchical clustering. This particular clustering method defines the cluster distance between two clusters to be the maximum distance between their individual components. At every stage of the clustering process, the two nearest clusters are merged into a new cluster. The process is repeated until the whole data set is agglomerated into one single cluster. Two distances were used: euclidean and Pearson correlation.

3.1 Clustering Analysis, euclidean distance

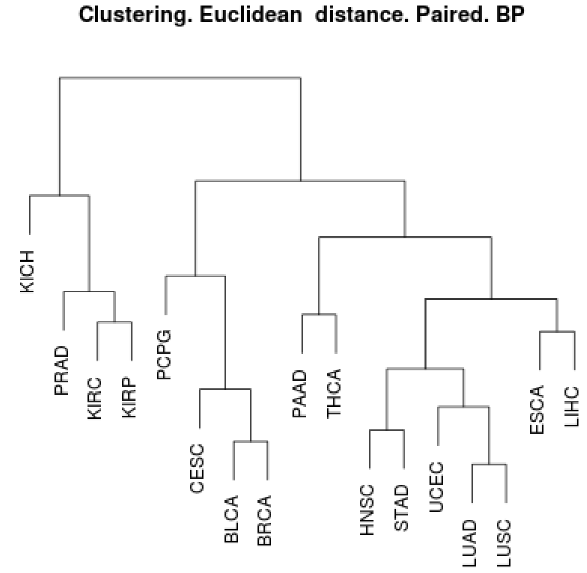

Figure 8: Clustering from enrichment results (Biological Processes), euclidean distance. TCGA paired studies

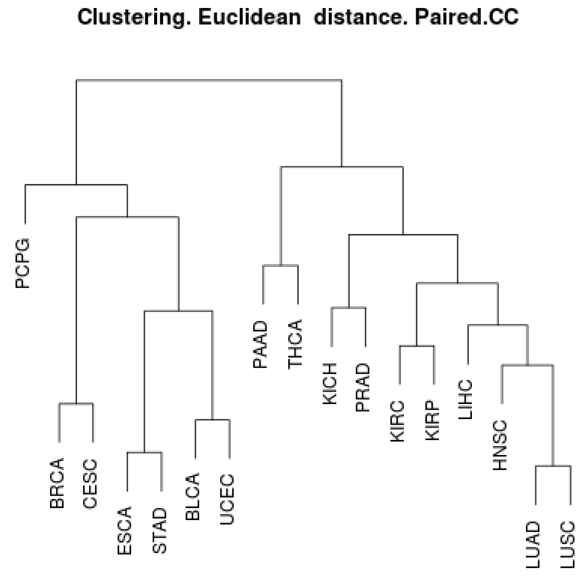

Figure 9: Clustering from enrichment results (Cellular Components), euclidean distance. TCGA paired studies

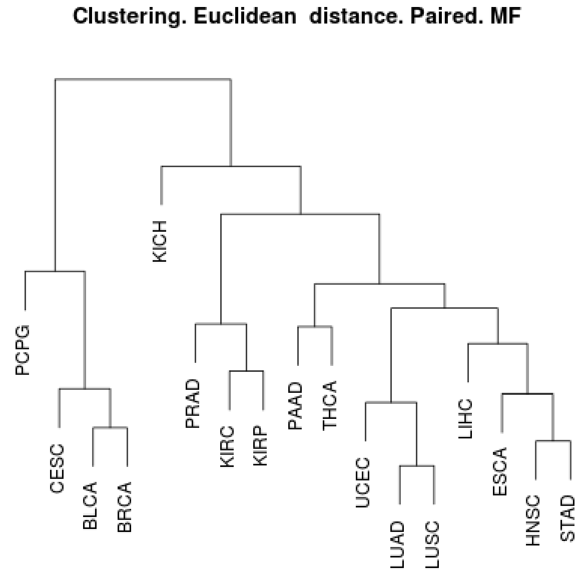

Figure 10: Clustering from enrichment results (Molecular Functions), euclidean distance. TCGA paired studies

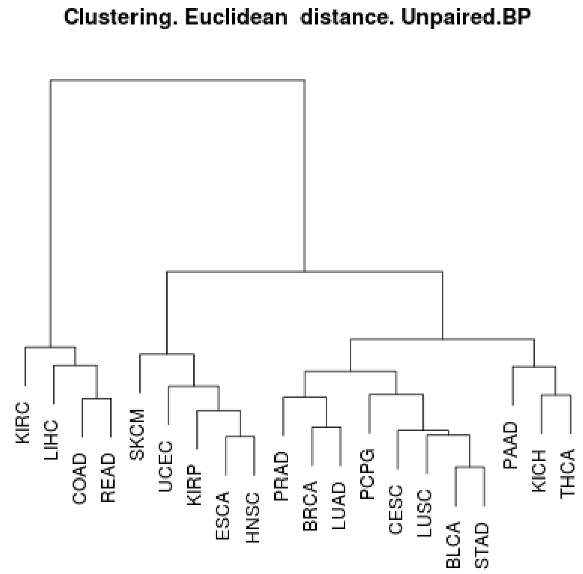

Figure 11: Clustering from enrichment results (Biological Processes), euclidean distance. TCGA unpaired studies

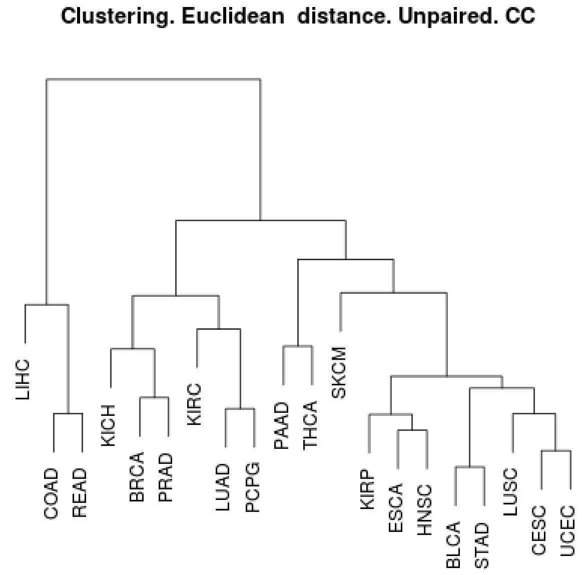

Figure 12: Clustering from enrichment results (Cellular Components), euclidean distance. TCGA unpaired studies

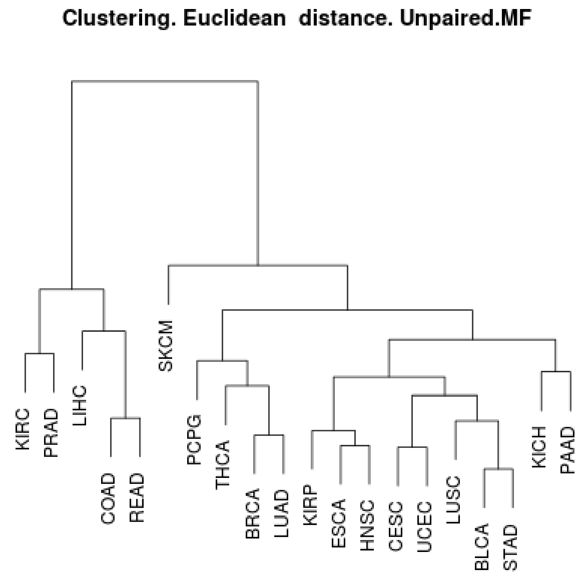

Figure 13: Clustering from enrichment results (Molecular Functions), euclidean distance. TCGA unpaired studies

3.2 Clustering Analysis, correlation distance

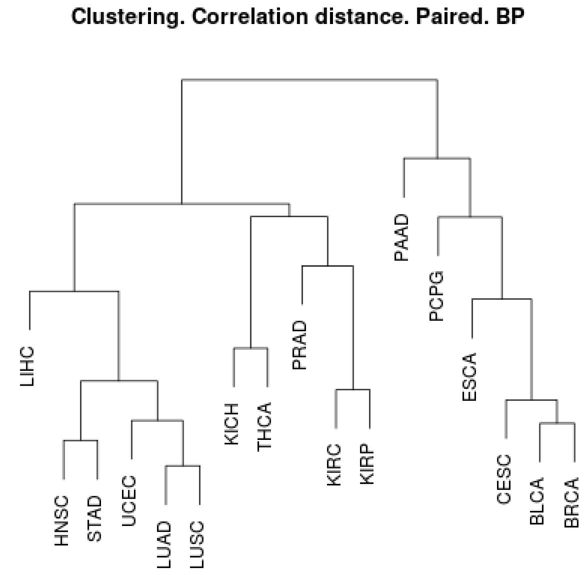

Figure 14: Clustering from enrichment results (Biological Processes), correlation distance. TCGA paired studies

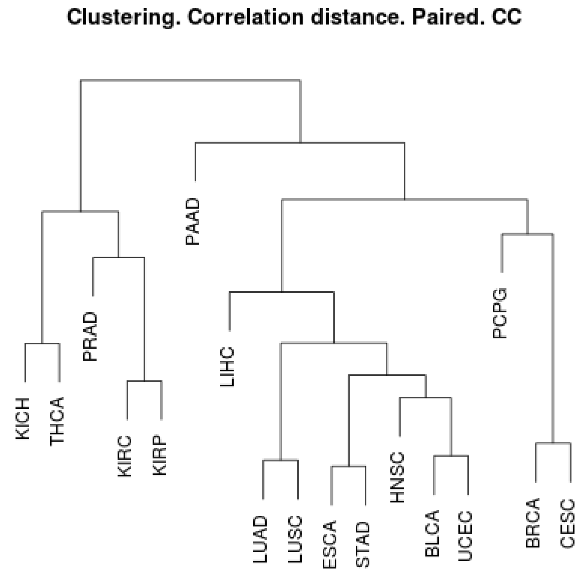

Figure 15: Clustering from enrichment results (Cellular Components), correlation distance. TCGA paired studies

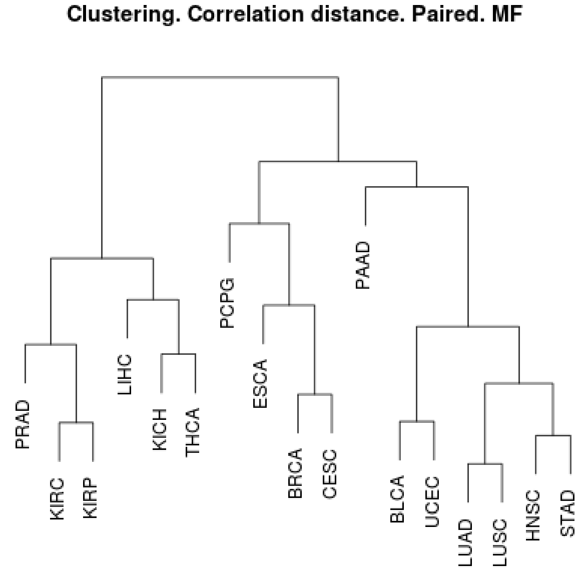

Figure 16: Clustering from enrichment results (Molecular Functions), correlation distance. TCGA paired studies

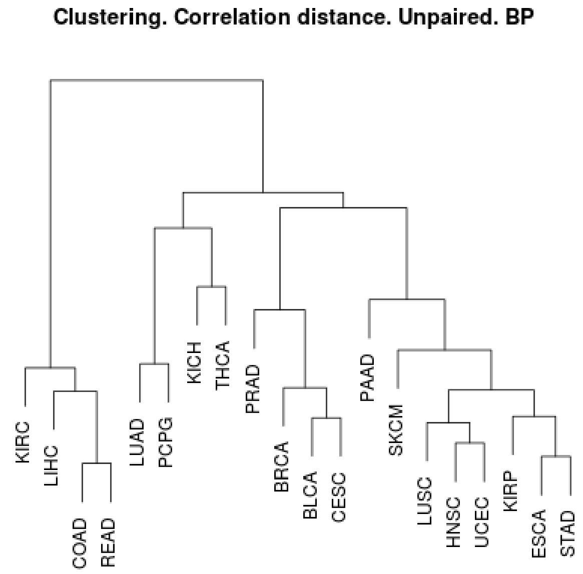

Figure 17: Clustering from enrichment results (Biological Processes), correlation distance. TCGA unpaired studies

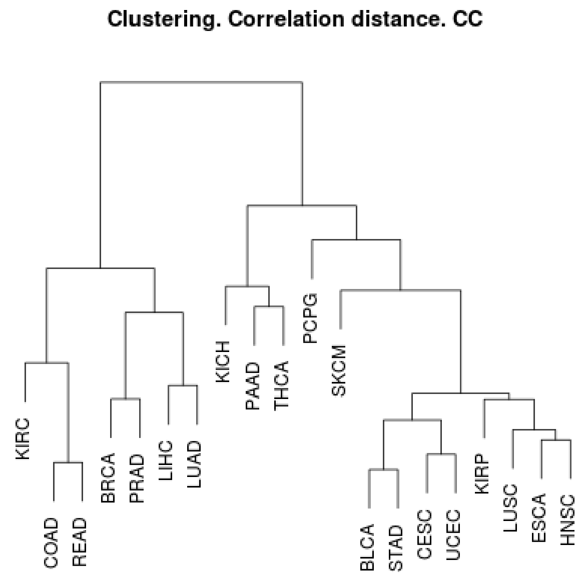

Figure 18: Clustering from enrichment results (Cellular Components), correlation distance. TCGA unpaired studies

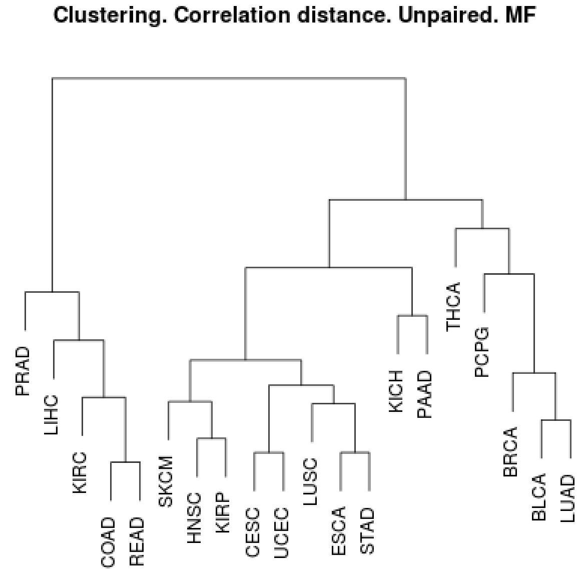

Figure 19: Clustering from enrichment results (Molecular Functions), correlation distance. TCGA unpaired studies

3.3 Significant Clustering Analysis, correlation distance

Cluster analysis was evaluated from **pvclust** (`http://www.sigmath.es.osaka-u.ac.jp/shimo-lab/prog/pvclust/`). This R package calculates probability values (p-values) for each cluster using bootstrap resampling techniques.

P-value of a cluster is a value between 0 and 1, which indicates how strong the cluster is supported by data. **pvclust** provides two types of p-values: AU (Approximately Unbiased) p-value and BP (Bootstrap Probability) value. AU p-value, which is computed by multiscale bootstrap resampling, is a better approximation to unbiased p-value than BP value computed by normal bootstrap resampling.

pvclust performs hierarchical cluster analysis via function hclust and automatically computes p-values for all clusters contained in the clustering of original data. It also provides graphical tools such as plot function or useful pvrect function which highlights clusters with relatively high/low p-values.

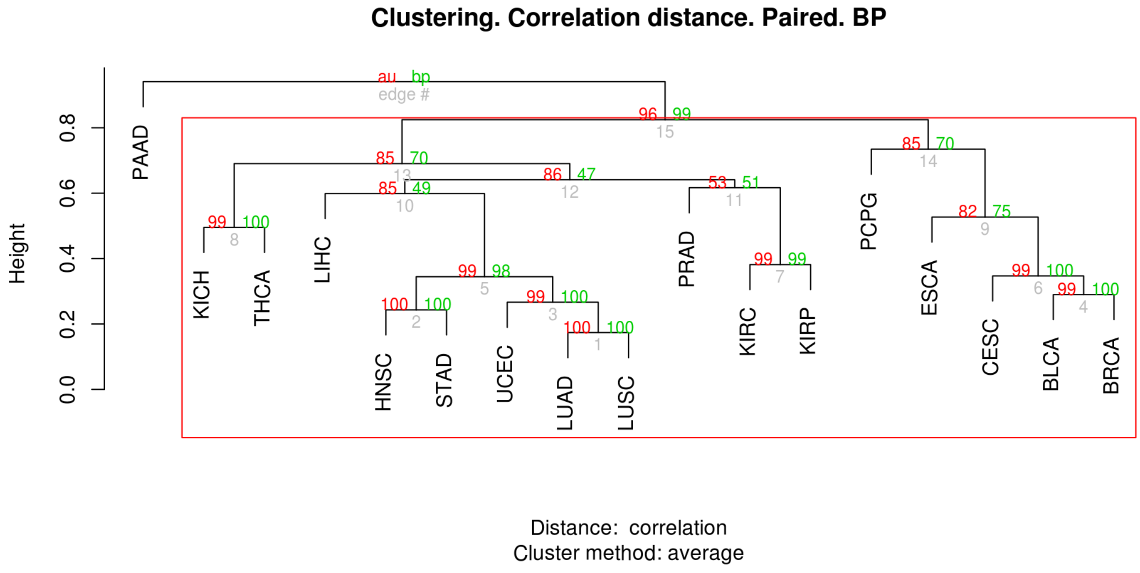

Figure 20: Significant clustering from enrichment results (Biological Processes), correlation distance. TCGA paired studies

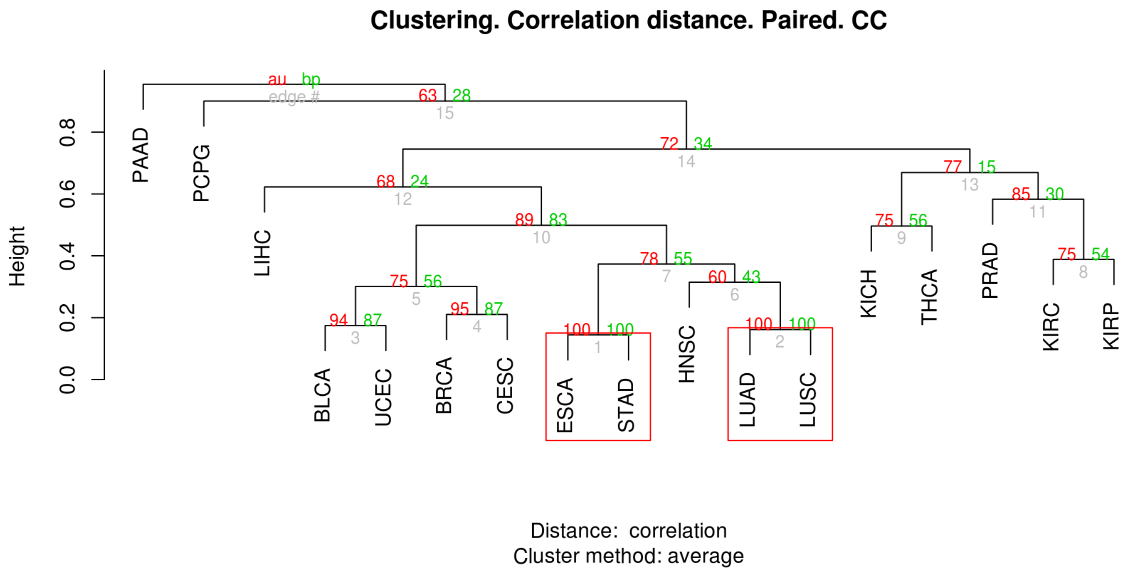

Figure 21: Significant clustering from enrichment results (Cellular Components), correlation distance. TCGA paired studies

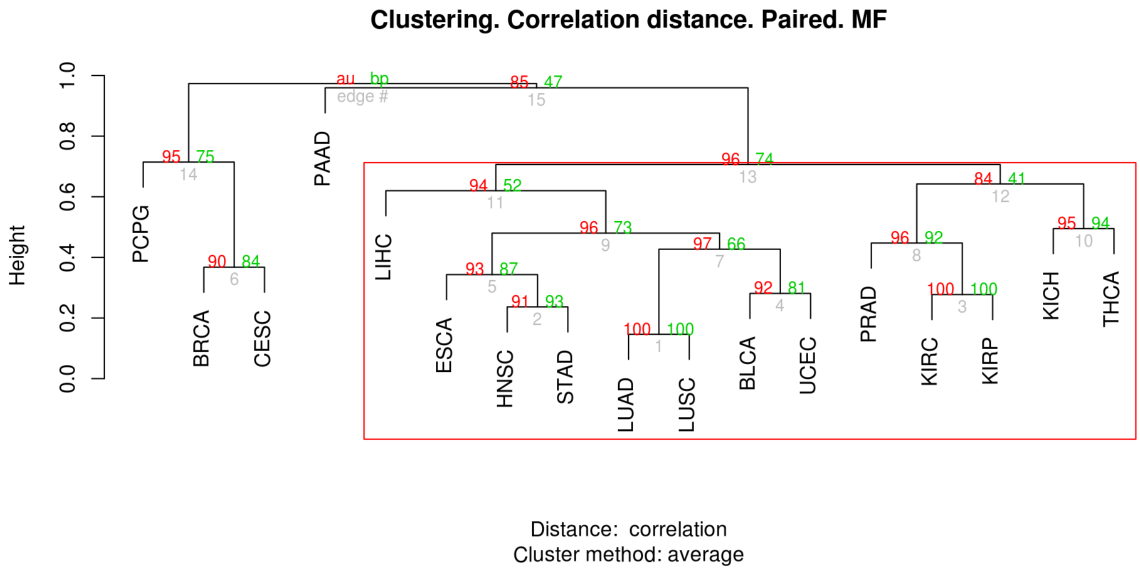

Figure 22: Significant clustering from enrichment results (Molecular Functions), correlation distance. TCGA paired studies

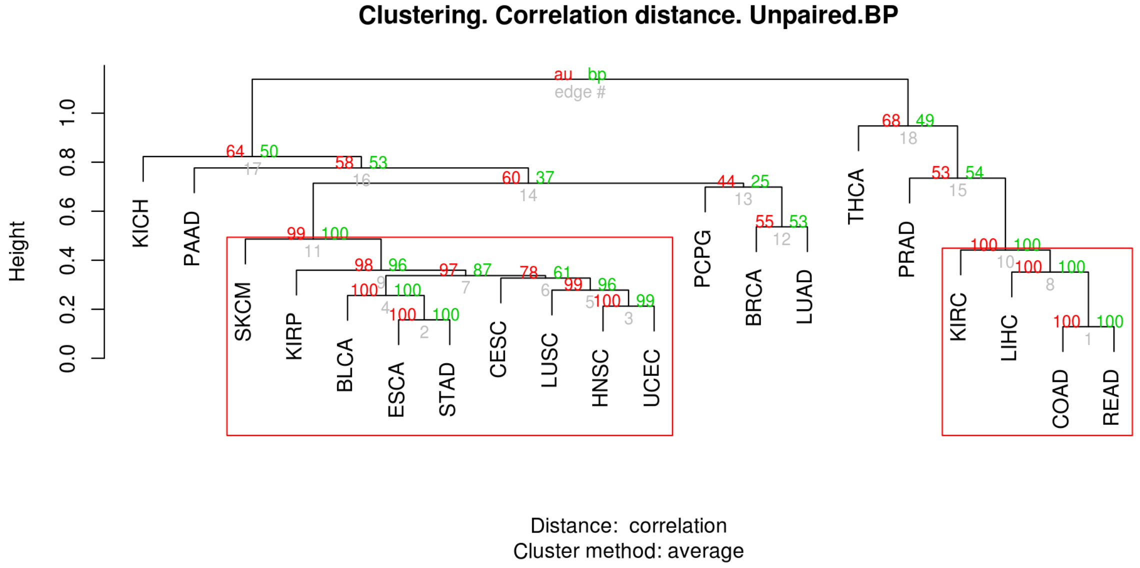

Figure 23: Significant clustering from enrichment results (Biological Processes), correlation distance. TCGA unpaired studies

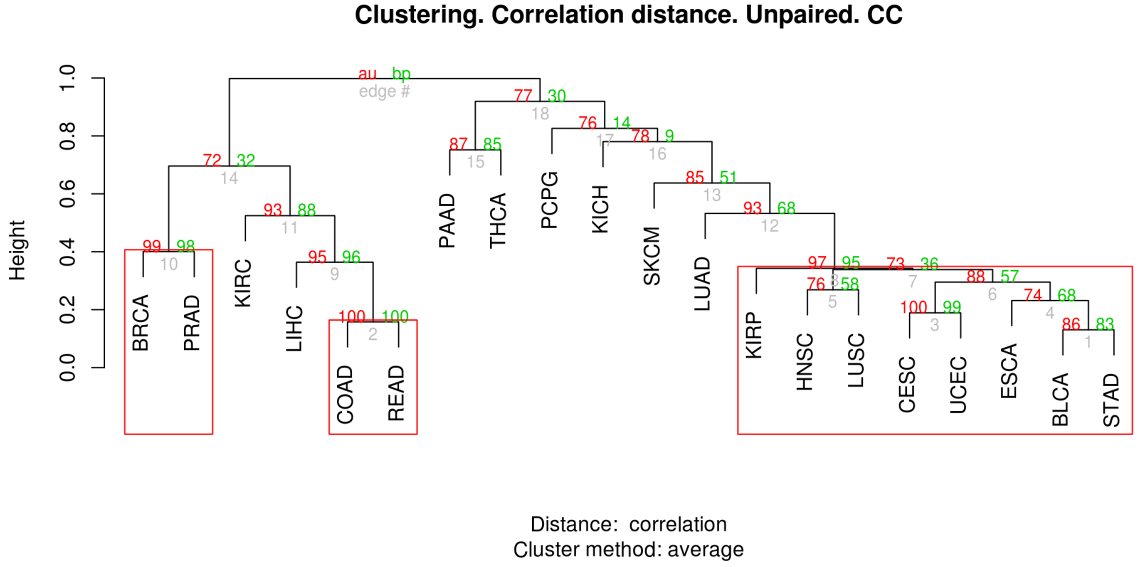

Figure 24: Significant clustering from enrichment results (Cellular Components), correlation distance. TCGA unpaired studies

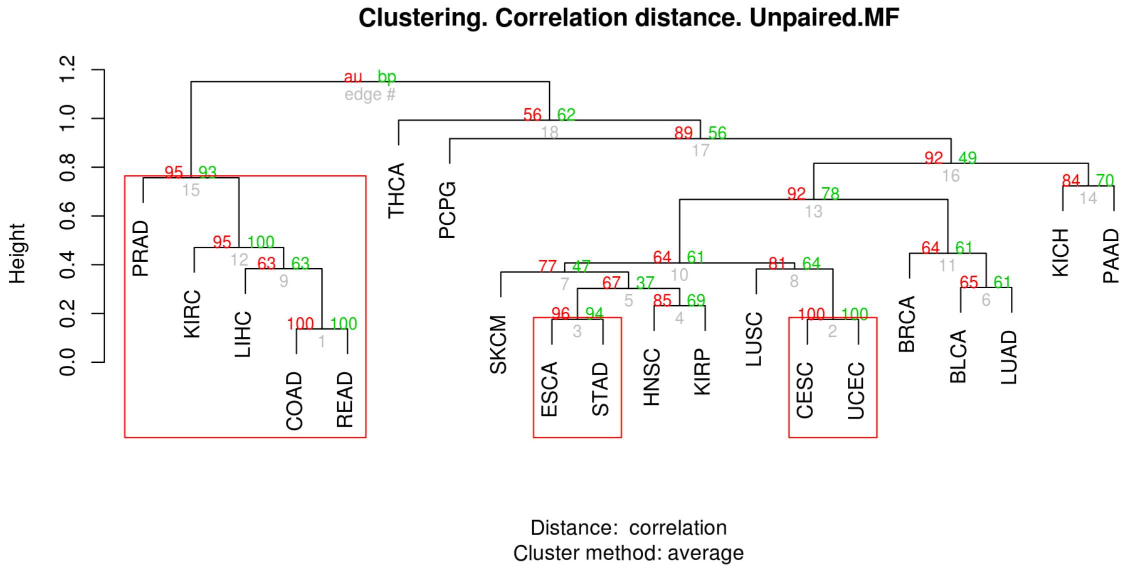

Figure 25: Significant clustering from enrichment results (Molecular Functions), correlation distance. TCGA unpaired studies

References

- Everitt, B. (1974). Cluster Analysis. London: Heinemann Educ. Books.
- Suzuki R and Shimodaira H (2006). Pvclust: an R package for assessing the uncertainty in hierarchical clustering. Bioinformatics. Jun 15;22(12):1540-2. Epub 2006 Apr 4.
- Montaner D and Dopazo J (2010). Multidimensional Gene Set Analysis of Genomic Data. PLoS One, 5(4), pp. e10348.
