# Supplementary material for: Integrated gene set analysis for microRNA studies
Source: Bioinformatics. 2016 Jun 20;32(18):2809–16. doi: 10.1093/bioinformatics/btw334 (PMC5018374; doi:10.1093/bioinformatics/btw334)
Supplement: Supplementary Data [file supp_btw334_suppl_data.zip › expressed_genes_report.pdf]

Integrated Gene Set Analysis for microRNA Studies

Analyzing just expressed genes

2016-05-17

Introduction

In our paper we present methods and software for the functional interpretation of microRNA expression data on its own. But many times, extra information such as gene expression levels may be also available. In such cases, researchers may want to restrict their functional interpretation just to those target genes which are effectively expressed. This sounds sensible as mRNA bridges miRNA functionality.

In this supplementary document we show how such analysis can be trivially done using our `mdgsa` library.

Methods

When a list of expressed genes is available we can modify equation (2) of the main paper as follows:

$$t_i = \begin{cases} \sum_{j \in G_i} r_j & \text{if gene } i \text{ is expressed} \\ 0 & \text{if gene } i \text{ is not expressed} \end{cases}$$

This modification in the formula does not change our approach but accounts for the non expression of the miRNA target genes.

Code

In this section we provide the commands from the `mdgsa` library used to carry out the functional interpretation of the microRNA expression data. We indicate how they should be modified to exclude non expressed genes as indicated in the above modification of equation (2).

Let p and s indicate respectively the *p-values* and the *test statistics* resulting form the differential expression analysis of the miRNA dataset. These will be R vectors.

Firs we will summarize the two quantities as indicated in **equation (1)**. This can be done using the function `pval2index` in the library:

```
index.mirna.level <- pval2index (pval = p, sign = t)
```

Then we will *transfer* the index from miRNA to mRNA level as indicated in **equation (2)**. This can be done using the function `transferIndex`:

```
index.gene.level <- transferIndex (index.mirna.level, targets)
```

in this steps `targets` is an R *list* containing gene targets for each microRNA.

Up to here this is the standard implementation of our methodology. Now, if we have a list of expressed genes in an R vector `EG`, we can set to 0 the values in `index.gene.level` corresponding to genes outside `EG` using the following command:

```
index.gene.level <- index.gene.level * names (index.gene.level) %in% EG
```

This last trivial step will effectively transform equation (2) as indicated above, setting to 0 all *transferred* miRNA effects in non expressed genes.

Then, the final step of the *gene set analysis* can be carried out as proposed in **equation (3)** using the function `uvGsa`:

```
res <- uvGsa (index.gene.level, annot)
```

where `annot` is a list containing the gene annotation.

Notice that just the third command

```
index.gene.level <- index.gene.level * names (index.gene.level) %in% EG
```

is needed to account for the non expressed genes. The standard analysis when no information of the gene expression is available will still use the same three remaining steps as devised in the library:

```
index.mirna.level <- pval2index (pval = p, sign = t)
index.gene.level <- transferIndex (index.mirna.level, targets)
res <- uvGsa (index.gene.level, annot)
```

Results

As a usage example, the above described approach was taken to reanalyze the **KICH** (Kidney Chromophobe) dataset in TCGA.

Normalized Gene expression levels where downloaded from the TCGA web site. Genes where considered expressed if they had normalized counts above 1 for all of the samples in the study. With this, 8821 (73%) genes of the 12084 targeted by miRNAs where described as expressed. The functional interpretation was carried out as described in the methods section. All scripts used for the analysis can be found in:
https://github.com/dmontaner-papers/gsa4mirna/tree/gh-pages/scripts_expression

Overall, correlation between results of the functional interpretation with and without the gene expression consideration was 0.5 for the *paired* analysis and 0.41 for the *unpaired* one. Both significant with a 0.05 confidence level.

Few GO terms such as *homophilic cell adhesion via plasma membrane adhesion molecules* (GO:0007156) and *histone modification* (GO:0016570) resulted significant in both analyses, while many others where significant just when considering or not the expressed genes. This may be a good indicator of the fundamental differences between the two approaches.

All results of the analysis considering the expressed genes are available in:
https://github.com/dmontaner-papers/gsa4mirna/tree/gh-pages/supplementary_files_expression
